# Broadband Achromatic Programmable Electromagnetic Camouflage via Fluidic‐Accessible Metasurface

**DOI:** 10.1002/advs.202523971

**Published:** 2026-01-09

**Authors:** Shipeng Liu, Jiahao Zhang, Jiale Wang, Zeyao Chen, Zhaotong Yu, Jinhong Luo, Jiabao Luo, Rujia Cao, Shuxi Gong, Qingxin Guo, Ping Li, Rujiang Li, Ke Chen, Yongtao Jia, Ying Liu

**Affiliations:** ^1^ School of Electronic Engineering Xidian University Xian China; ^2^ School of Information and Communication Engineering Communication University of China Beijing China; ^3^ Applied Physics Technology Center Beijing Institute of Mechanical Equipment Beijing China; ^4^ School of Electronic Science and Engineering Nanjing University Nanjing China

**Keywords:** achromatic strong scattering point, broadband and reconfigurable radar illusion, fluidic‐accessible metasurface, quasi‐3D microfluidic meta‐atom, solid–liquid metal hybrid architecture

## Abstract

Broadband and reconfigurable illusion camouflage remains a major challenge in electromagnetic wave manipulation, as it imposes concurrent demands for precise dispersion control together with reliable real‐time switching across wide frequency ranges. Existing metasurface cloaks typically suffer from narrow bandwidths and limited adaptability, rendering them unsuitable for dynamic radar detection scenarios. Herein, we propose and experimentally demonstrate a Fluidic‐Accessible Metasurface (FAM) that overcomes these limitations by enabling programmable electromagnetic illusions through focal‐spot encoding. The supercells are designed with strong dispersion control and achromatic focusing capabilities, thereby generating stable scattering hotspots from the radar perspective. Through the assembly and fluidic reconfiguration of these supercells, the FAM dynamically reconstructs the illusionary contours of diverse targets, such as aircraft, drones, and tanks, within an ultrawide operational bandwidth of 9–14 GHz. Experimental near‐field measurements, in agreement with full‐wave simulations, verify reliable and repeatable illusion camouflage states without leakage or degradation during fluidic reconfiguration. This strategy, therefore, unifies broadband operation, dynamic programmability, high adaptability, and structural robustness, directly addressing the key limitations of existing metasurface cloaks. This work establishes a versatile platform for programmable electromagnetic illusions, enabling the practical deployment of next‐generation intelligent metasurface camouflage systems.

## Graphical abstract

A fluidic access metasurface enables dynamic, broadband electromagnetic illusions. By physically reconstructing conductive patterns with liquid metal, the metasurface achieves real‐time, pixel‐level control over phase dispersion. This allows for the programmable generation of arbitrary strong scattering point arrays, effectively deceiving high‐resolution radar systems with convincing phantom targets.

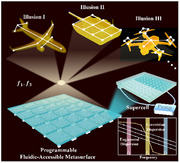

## 1 Introduction

Electromagnetic illusion, a compelling concept in countermeasures, seeks to manipulate scattered fields to alter a target's perceived identity or location. Achieving this fundamentally relies on precise wavefront tailoring to mimic a different scattering signature [1, 2, 3, 4, 5, 6, 7, 8, 9, 10]. The theory of transformation optics (TO) provides a rigorous framework for such wavefront control by engineering spatial variations in material parameters [1]. Building on this, strategies like conformal mapping and scattering cancellation have demonstrated the feasibility of electromagnetic camouflage [4]. However, the practical application of these TO‐based approaches is severely constrained by stringent material requirements. The demand for spatially‐variant, often anisotropic constitutive parameters culminates in complex, 3D architectures [6]. Consequently, these cloaks are characteristically bulky, narrowband, and static, limitations that have significantly hindered their scalability and adaptability for real‐world scenarios.

Metasurfaces represent a landmark innovation for wave control, capable of imparting arbitrary phase profiles upon incident wavefronts by engineering meta‐atoms [11, 12, 13, 14, 15, 16, 17, 18, 19, 20, 21, 22, 23, 24, 25, 26, 27, 28, 29, 30, 31]. This has unlocked unprecedented potential for electromagnetic camouflage. Various metasurfaces have been proposed to achieve broadband illusions, yet these pioneering works are typically static [32]. For instance, while ultra‐broadband cloaking has been demonstrated, the designs preclude post‐fabrication adaptability [32]. Similarly, other strategies offer only a discrete set of predefined camouflage states [33]. To address the camouflage of intricate targets within this static framework, a dispersion‐modulated multi‐focal encoding strategy has recently emerged [34]. This approach enables broadband achromatic focusing to camouflage complex targets like aircraft by satisfying the required phase profiles over a wide frequency range. Despite this significant progress, the practical implementation of these advanced camouflage devices remains fundamentally static, lacking the crucial capability for real‐time adaptation.

The pursuit of dynamic functionality has driven the integration of active components into metasurfaces, yet established tuning mechanisms present a collective impasse. Electronic tuning with PIN or varactor diodes, while fast, suffers from significant insertion losses and requires complex biasing networks that distort the scattered fields, limiting precise broadband phase control [35, 36]. Mechanical reconfiguration via MEMS offers a low‐loss alternative but is hindered by high actuation voltages, environmental sensitivity, and fabrication complexity [37, 38]. Phase‐change materials like Vanadium Dioxide (VO_2_) provide a distinct state change but are constrained by cycling fatigue and reliance on external stimuli, offering limited flexibility beyond a fixed geometric layout [39, 40]. Furthermore, the power handling capability of these elements is a critical bottleneck for high‐power microwave applications. For instance, while specialized PIN diodes can manage up to 35–50 W and RF MEMS can handle up to 25 W, they still face challenges such as thermal effects or reliability issues under continuous high‐power operation. Crucially, while these established methods provide reconfigurability, they all operate by tuning the properties of components within a geometrically static layout. They enable localized parameter changes but lack the capacity for true, arbitrary physical reconstruction of the active conductive elements. This inherent architectural constraint is the root cause of the trade‐offs that preclude the simultaneous achievement of broadband operation, low loss, and robust dispersion control, all of which are essential for high‐fidelity, dynamic electromagnetic illusions.

To address the aforementioned architectural constraint, liquid metals (LMs) have emerged as a compelling class of reconfiguration elements. Gallium‐based eutectic alloys are liquid at room temperature and possess metallic conductivity, ensuring inherently low‐loss performance and superior power handling capabilities that circumvent the limitations of semiconductor devices [25]. Their defining feature is the capacity for true geometric reconstruction within microfluidic channels, fundamentally altering the physical layout of conductive elements rather than merely modulating properties within a fixed structure. This unique capability enables unparalleled versatility, allowing a single array to be dynamically reconfigured for diverse functions. Capitalizing on these properties, research has demonstrated various LM‐integrated devices, such as reconfigurable antennas and filters [26, 41, 42, 43, 44, 45, 46, 47]. However, a critical review reveals that these works primarily focus on tuning discrete operational parameters like resonant frequency or beam direction. The dynamic, spatially‐resolved control of a more fundamental electromagnetic property, phase dispersion, through arbitrary geometric reconfigurability remains a largely unexplored frontier. This capability is paramount for advanced wavefront shaping, as it directly governs the group delay of propagating waves and is the key to achieving the broadband, achromatic functionalities essential for high‐fidelity illusion camouflage.

Herein, we bridge this critical gap by proposing and experimentally demonstrating a Fluidic‐Accessible Metasurface (FAM) that integrates LM to achieve simultaneous broadband and dynamically reconfigurable electromagnetic illusions. Our platform, based on a hybrid solid–liquid metal design, uniquely enables the dynamic, pixel‐level control of phase dispersion, a capability previously unattainable with conventional active tuning mechanisms. By precisely engineering the dispersion of quasi‐3D microfluidic meta‐atoms, the FAM's supercells achieve broadband achromatic focusing. This allows for the real‐time, targeted injection and withdrawal of LM within embedded channels, which reconfigures these achromatic focal distributions in 3D space. The result is the formation of programmable strong scattering point (SSP) arrays capable of generating complex illusion profiles on demand. Comprehensive experimental and numerical results validate the effectiveness, fidelity, and adaptability of this approach. By demonstrating programmable control over a fundamental wave property through physical reconstruction, our work establishes a new paradigm for real‐time, spatially reconfigurable EM illusion devices and offers significant potential for next‐generation adaptive camouflage and stealth technologies.

## 2 Results and Discussion

### 2.1 Operating Principle

The fundamental principle behind our proposed illusion camouflage hinges on the dynamic manipulation of SSPs, which are the primary determinants of a target's signature in high‐resolution radar imaging systems like Synthetic Aperture Radar (SAR). These SSPs typically arise from physical discontinuities on the target, such as edges or corners, where the local radar cross‐section (RCS) is maximized. Consequently, the ability to arbitrarily control the spatial and spectral properties of these scattering points provides a powerful means for generating high‐fidelity electromagnetic illusions.

Crucially, for a synthesized broadband radar image, if the scattering centers are chromatic, the resulting SSPs will be spatially smeared, leading to a blurred and distorted image that fails to generate a convincing illusion. Therefore, to create sharp, believable illusionary targets, it is imperative to engineer scattering centers that maintain a stable spatial position across the entire operational bandwidth—a property known as achromatic focusing.

**FIGURE 1:**
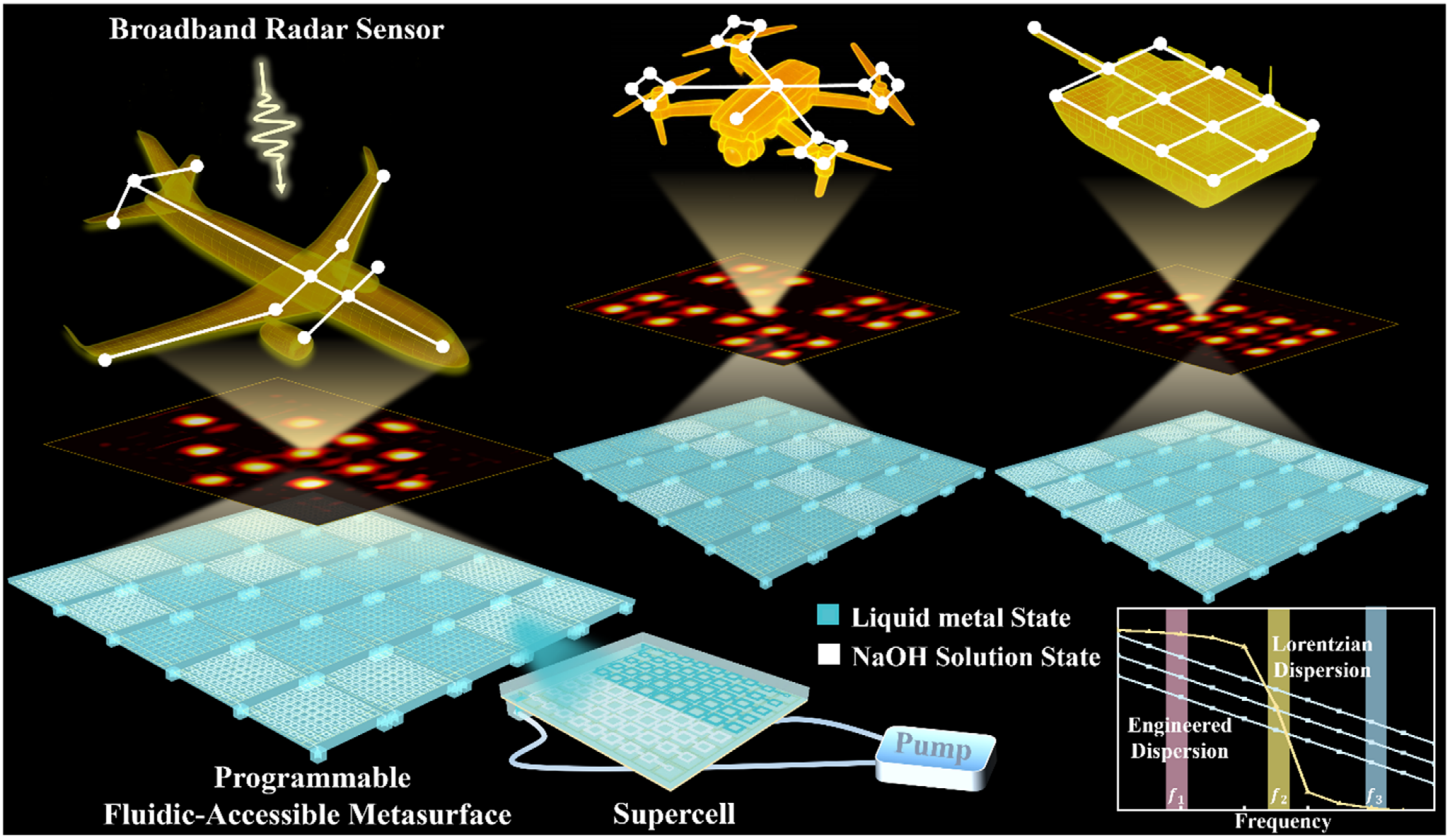
Schematic illustration of the reconfigurable electromagnetic illusion enabled by the FAM. The main panel depicts the FAM platform achieving adaptive radar camouflage for various complex targets. By dynamically programming the liquid filling states within its microfluidic channels, the FAM can generate arbitrary illusionary scattering signatures under broadband electromagnetic wave illumination. Specifically, an external multi‐channel pumping system is employed to independently regulate the fluidic state of individual pixels, ensuring precise and flexible control. The inset in the bottom right corner illustrates the core principle of dispersion engineering. It shows the stark contrast between the sharp, non‐linear Lorentzian dispersion of a conventional meta‐atom and the highly linear phase response provided by our engineered meta‐atom across the entire operational bandwidth. This engineered linear dispersion is the fundamental requirement for achieving such broadband, achromatic functionalities.

As schematically illustrated in Figure 1, our proposed FAM is designed to achieve precisely this goal. The FAM consists of an array of programmable supercells, each engineered to function as an independent, achromatically focusing lens. This is achieved by meticulously tailoring the phase‐dispersion relationship of each constituent meta‐atom. This requirement for independent, frequency‐by‐frequency control over the meta‐atom's phase response is the key to ensuring consistent focusing behavior, a formidable challenge that our FAM platform is designed to overcome.

### 2.2 Meta‐Atom Design

#### 2.2.1 Static Dispersion Engineering via Coupled Resonators

**FIGURE 2:**
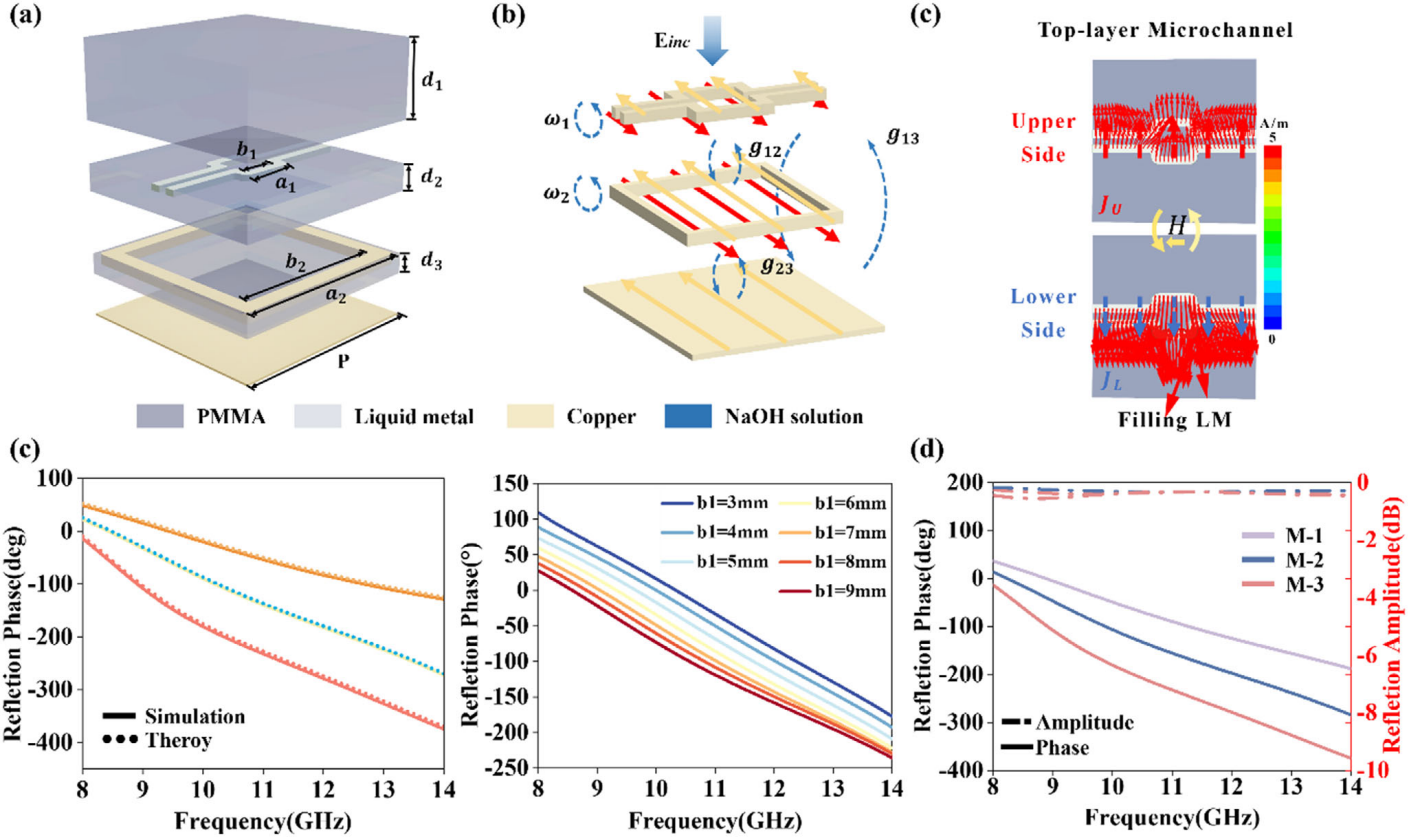
Static dispersion engineering framework and design flexibility of the meta‐atom. (a) Exploded view of the multi‐layered meta‐atom structure. (b) Equivalent coupled resonator model for the ON state, illustrating self‐resonances (ω_*n*_) and inter‐layer couplings (*g_mn_*). (c) Simulated surface current distribution in the ON state, indicative of strong magnetic resonance. (d) Excellent agreement between the simulated reflection phase (solid line) and the coupled Lorentz oscillator model (dashed line). (e) Systematic control over phase dispersion demonstrated by a parametric sweep of the middle ring's side length b_1_. The color map indicates the continuous tuning of the phase profile as the geometry is varied. (f) Resultant linear phase dispersion profiles for three representative meta‐atoms (M‐1, M‐2, M‐3), which form the basis of the static design library.

The realization of broadband, achromatic control necessitates a meta‐atom capable of providing a linear phase dispersion profile. To this end, we propose a quasi‐3D meta‐atom based on a solid–liquid metal hybrid architecture, as depicted in the exploded view in Figure 2a. The design consists of three metallic layers separated by a Polymethyl Methacrylate (PMMA, ε_*r*_ = 2.54 and tan δ = 0.0083) dielectric spacer: a top layer with a reconfigurable microfluidic channel, a middle layer made of a solid‐state metallic square ring, and a bottom continuous ground plane for total reflection.

Functionally, this multilayer structure operates as a coupled resonator system, which forms the physical basis for its advanced dispersion engineering capabilities. In its "ON" state, where the microfluidic channel is filled with LM (Galinstan, σ = 3.55 × 10^6^ S · m^−1^), the incident electromagnetic wave excites strong, anti‐parallel surface currents on the top LM pattern and the middle metallic ring. This is visually represented by the coupled resonator model and the surface current distributions shown in Figure 2b, respectively. The mutual interaction between these current paths establishes a cascade of near‐field couplings (*g*_12_, *g*_23_) and, critically, a direct through‐layer coupling (*g*_13_) that governs the overall response. The meta‐atom's reflection phase, φ(ω), can be effectively described by a coupled Lorentz oscillator model:

(1) $$\varphi \left(\omega \right)=arg\left[\sum_{n=1}^{N}\frac{A_{n}e^{i\theta_{n}}}{\omega^{2}-\omega_{n}^{2}+i\gamma_{n}\omega }\right]$$

where ω_*n*_ represents the resonant frequency, γ_*n*_ is the damping factor associated with its energy loss, *A_n_* quantifies the amplitude strength of the coupling, and θ_*n*_ is the initial phase of the *n^th^* magnetic mode. As demonstrated in Figure 2c, this theoretical model shows excellent agreement with the full‐wave simulation results for the ON‐state response, validating our physical understanding. (The design principles of the FAM are outlined in Note S3).

Crucially, this theoretical framework provides a direct pathway for static dispersion engineering. The physical geometries of the solid‐state metallic structures, specifically the dimensions of the top LM pattern and the middle square ring, serve as the knobs to control the parameters within the Lorentz model. By systematically and concurrently varying these geometric parameters, we can precisely tailor both the individual resonant frequencies (ω_*n*_) and the inter‐layer coupling strengths (*g_mn_*). This multi‐parameter optimization allows us to strategically shape the overall phase response φ(ω) and engineer a near‐arbitrary linear phase dispersion profile within the target bandwidth. Figure 2d exemplifies this powerful design capability. It presents three representative meta‐atoms (M‐1, M‐2, and M‐3), each exhibiting a distinct, highly linear phase response across the operational bandwidth. These customized dispersion profiles are achieved simply by modifying the inner and outer dimensions of the solid‐state square rings. This static engineering process, rooted in the strategic manipulation of a multi‐resonant system, enables precise, decoupled control over phase and group delay. It allows for the creation of a comprehensive library of meta‐atoms, each with a custom‐tailored linear dispersion profile, which are the essential building blocks for arbitrary broadband achromatic metasurface design.

#### 2.2.2 Dynamic Reconfiguration via Fluidic Access

**FIGURE 3:**
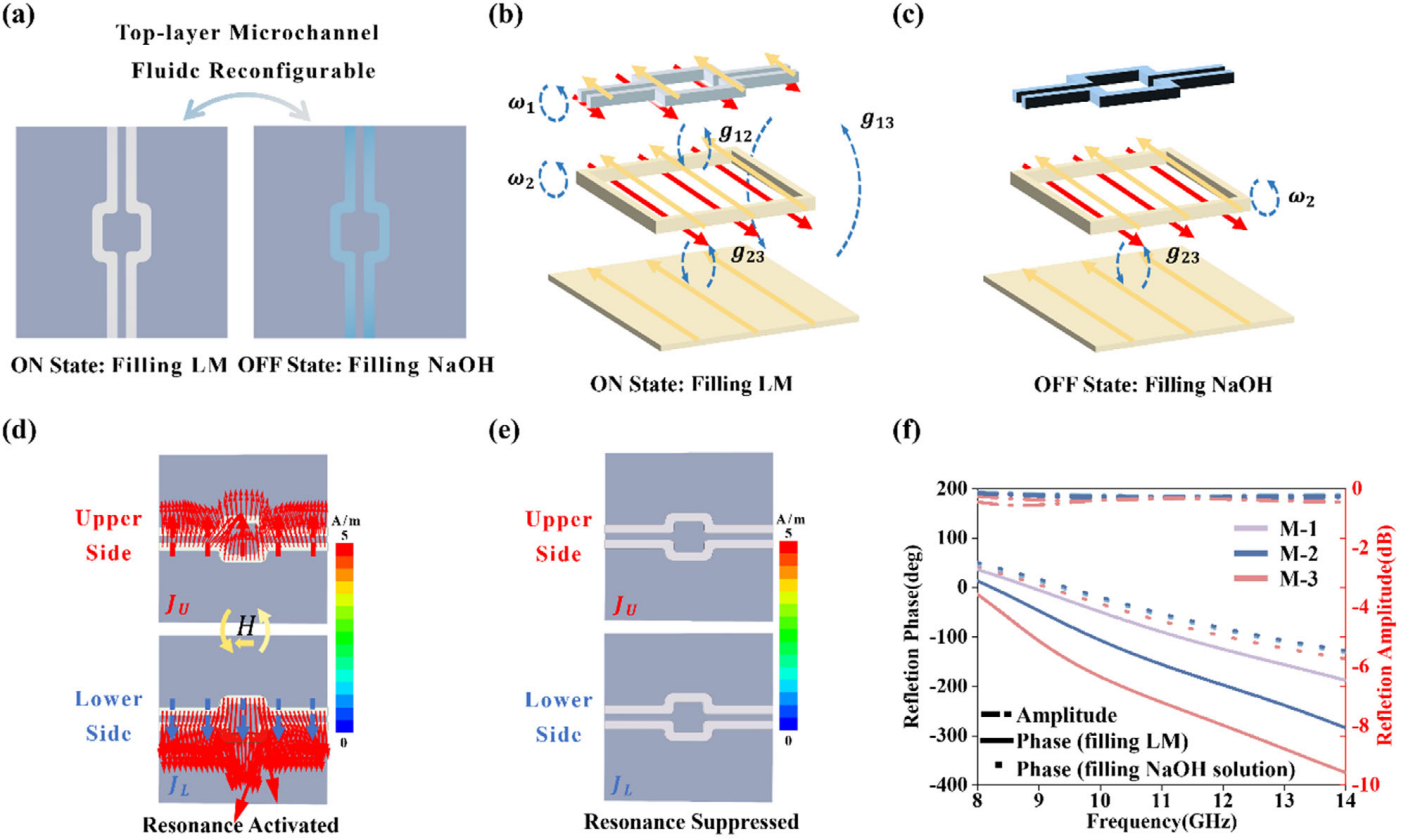
Dynamic reconfiguration mechanism and performance contrast. (a) Schematic illustration of the fluidic switching between the ON state (LM‐filled) and the OFF state (NaOH‐filled). (b, c) Equivalent coupled resonator models for the (b) ON state and (c) OFF state, illustrating the deactivation of the top‐layer resonator. (d, e) Comparison of the simulated surface current distributions in the (d) ON state and (e) OFF state, showing significant suppression of resonance. (f) Comparison of the reflection phase for three representative meta‐atoms (M‐1, M‐2, M‐3) in its ON state (linear dispersion) and OFF state (flat response), demonstrating high‐contrast dynamic switching.

Building upon the versatile static dispersion framework, we introduce a dynamic degree of freedom through fluidic reconfiguration. The ability to switch the meta‐atom between two distinct functional states stems from the selective infusion of different liquids into the top‐layer microchannel, as schematically illustrated in Figure 3a. The physical mechanism of this switching is a profound alteration of the electromagnetic boundary conditions, which can be precisely described by modifying the coupled Lorentz oscillator model itself. The state‐dependent complex reflection coefficient, *r*(ω, *S*), where *S* represents the fluidic medium, can be expressed as:

(2) $$\begin{array}{ccc} & & r\left(\omega ,S\right)\\ & & \;=\left\{\begin{array}{cc} \frac{A_{1}e^{i\theta_{1}}}{\underset{\mathrm{LM}-\mathrm{activated}\;\mathrm{mode}}{\omega^{2}-\omega_{1}^{2}+i\gamma_{1}\omega }}+\frac{A_{2}e^{i\theta_{2}}}{\underset{\mathrm{Shared}\;\mathrm{mode}}{\omega^{2}-\omega_{2}^{2}+i\gamma_{2}\omega }}+r_{bg} & if\;S=LM\\ \frac{A_{2}e^{i\theta_{2}}}{\underset{\mathrm{Shared}\;\mathrm{mode}}{\omega^{2}-\omega_{2}^{2}+i\gamma_{2}\omega }}+r_{bg} & if\;S=NaOH \end{array}\right\} \end{array}$$

This formulation explicitly illustrates the switching mechanism. The equivalent coupled resonator models for both states are presented in Figure 3b,c, visually summarizing the change in the system's resonant structure. In the “ON” state (*S* = *LM*), the conductive LM activates a powerful, low‐frequency magnetic resonance (ω_1_). This introduces a dominant “LM‐activated mode” into the system's response, which, through its coupling (*g*_12_, *g*_13_, *g*_23_)with the shared mode (ω_2_) from the solid‐state ring, dictates the overall engineered dispersion φ_*ON*_(*w*) = arg[*r*(*w*, LM)]. This is evidenced by the intense surface currents shown in Figure 3d. Conversely, in the “OFF” state (S = NaOH), the electrically neutral fluid suppresses the top‐layer resonance. This effectively removes the “LM‐activated mode” (ω_1_ term) from the summation. The system's response is then simplified, dominated solely by the weaker, higher‐frequency shared mode (ω_2_) associated with the middle ring. This results in a flat, non‐dispersive phase response φ_0*FF*_(*w*) = *arg*[*r*(*w*, *NaOH*)] and drastically diminished surface currents, as shown in Figure 3e. The term *r_bg_* represents the non‐resonant background reflection. This high‐contrast control, achieved by physically adding or removing a fundamental resonant term via fluidic actuation, is the cornerstone of our dynamic reconfigurability. Importantly, this switching capability is universally applicable to the entire library of meta‐atoms established in the static design phase. Figure 3f powerfully illustrates this effect by comparing the reflection phase of three representative meta‐atoms (M‐1, M‐2, M‐3) in its two states: a highly linear, dispersive profile in the ON state versus a flat, non‐dispersive response in the OFF state.

Ultimately, this hybrid architecture provides two essential and orthogonal degrees of freedom: a static framework defined by the fixed solid‐state geometry for establishing a library of customized linear dispersions, and a dynamic reconfiguration capability enabled by fluidic access for switching these functionalities on and off. This dual‐control mechanism makes the proposed meta‐atom a powerful and versatile building block for the reconfigurable achromatic supercells detailed in the following section. (A comprehensive database of all designed meta‐atoms is provided in S4).

### 2.3 Supercell Design

Building upon the unique capabilities of the meta‐atom, the next critical step is to assemble these elements into a functional aperture, known as the supercell. The ultimate goal of our design is to dynamically generate and manipulate SSPs, which dominate a target's radar signature. A supercell capable of broadband achromatic focusing provides the fundamental means to create such an SSP at a specific location in space. The theoretical requirement for such focusing at a predefined focal point F is governed by the spatial phase profile:

(3) $$\varphi \left(r,f\right)=-\left[\frac{2\pi f}{c}\left(\sqrt{r^{2}+F^{2}}-F\right)\right]+\varphi_{0}\left(f\right)$$

where ***r*** denotes the radial distance from a point on the aperture to the geometric center of its corresponding supercell, and φ_0_(ω) is a global reference phase constant. More importantly, for achromatic performance, the group delay, τ, must be frequency‐invariant. Differentiating the phase profile yields the target group delay:

(4) $$\tau \left(r,f\right)=-\frac{d\varphi \left(r,f\right)}{df}=\frac{2\pi }{c}\left(\sqrt{r^{2}+F^{2}}-F\right)-\frac{d\varphi_{0}\left(f\right)}{df}$$

Our engineering challenge is thus to spatially arrange a discrete set of meta‐atoms across the supercell aperture such that their individual phase and group delay characteristics collectively approximate these continuous target functions.

To meet this challenge, we designed a supercell that serves as the fundamental building block for programmable electromagnetic illusion generation. Unlike conventional architectures where control might be exerted at the single element level, we define the independent tuning capability at the level of the supercell. Consequently, each supercell is composed of a 9×9 array of meta‐atoms that are connected to a single fluidic control port and actuated collectively. This hierarchical control strategy is implemented to satisfy two critical requirements. First, achieving broadband achromatic performance necessitates simultaneous and independent control over both phase response and group delay. While each meta‐atom is geometrically engineered to provide a specific local dispersion, the supercell must act as a collective functional meta‐atom to integrate these diverse spectral responses and fulfill the complex spatial phase profile required for focusing. Second, from an engineering perspective, defining the supercell as the minimum addressable pixel significantly enhances scalability by avoiding the prohibitive complexity of routing independent fluidic lines to every sub‐wavelength element.

**FIGURE 4:**
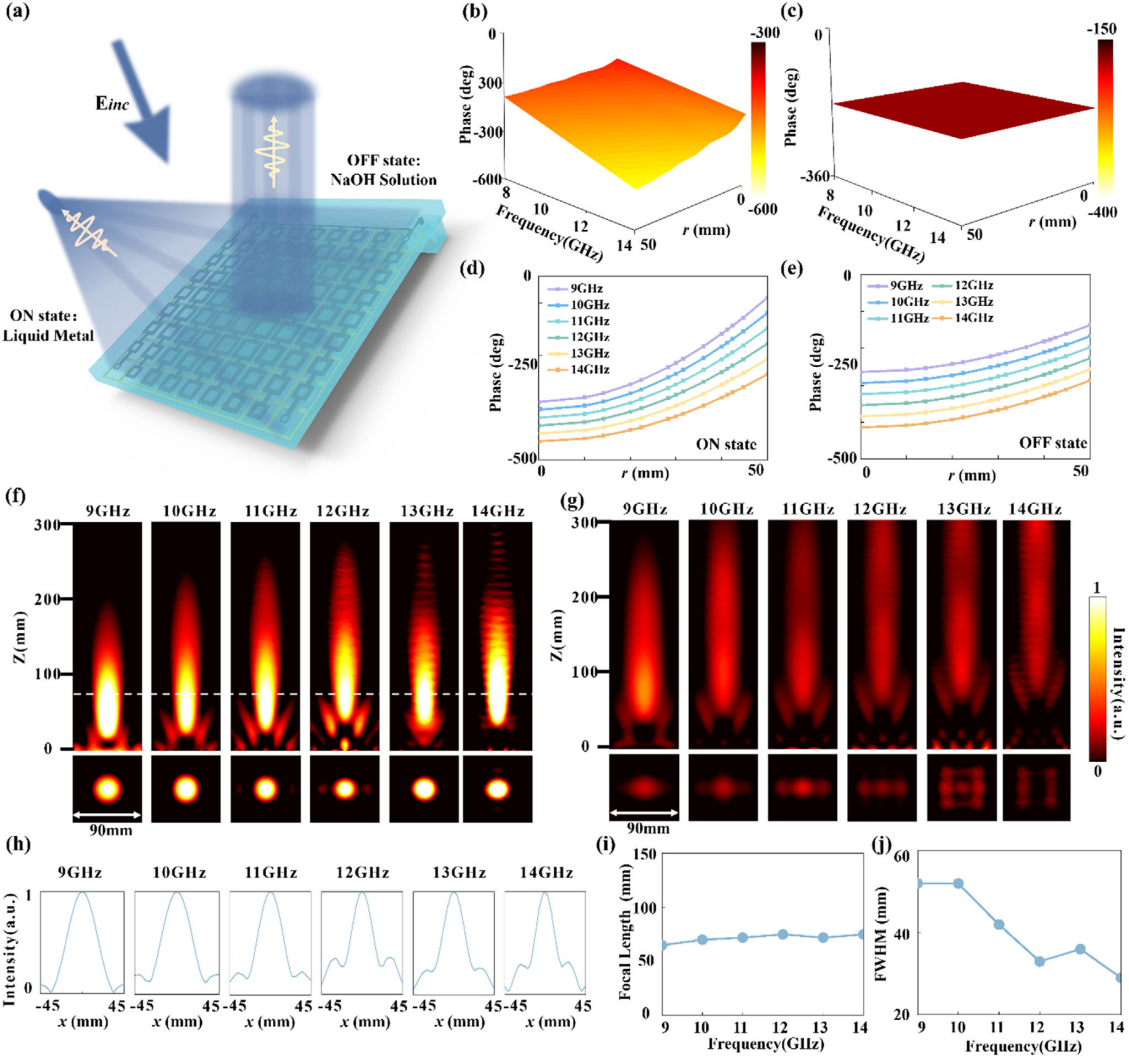
Design and performance validation of the reconfigurable achromatic focusing supercell. (a) Schematic of the supercell, composed of a 5×5 array of individually addressable meta‐atoms, designed to generate a SSP at the focal point F. (b, c) Target reflection phase profiles across the supercell aperture mapped as a function of radial distance *r* and frequency. b) The ON state exhibits a continuous, radially symmetric distribution required for broadband achromatic focusing, while c) the OFF state shows a near‐equiphase surface characteristic of planar reflection. (d, e) Required broadband reflection phase spectra for representative meta‐atoms located at different radial distances *r* from the geometric center of the individual supercell. In the (d) ON state, each meta‐atom exhibits a unique, linear dispersion profile tailored to its position. In the (e) OFF state, the phase spectra for all meta‐atoms s collapse onto a single, non‐dispersive flat line. (f, g) Simulated near‐field electric energy distributions. (f) The supercell in the ON state consistently focuses the wave at the target focal plane (F = 70 mm) across the 9‐14 GHz bandwidth. (g) The supercell in the OFF state acts as a simple reflector. Both maps share the same normalized color scale. f) Transverse normalized energy intensity in the focal plane located at the z position marked at the top left. g) Focal length and (h) FWHM across the X‐band.

This design paradigm results in a crucial distinction between spatial complexity and operational complexity. While the spatial phase distribution within the supercell is designed to be quasi‐continuous to ensure high‐efficiency wavefront shaping, the dynamic operation of the device remains strictly 1‐bit discrete. The microfluidic control system functions by toggling the LM between a fully filled Metallic State and an empty Dielectric State without intermediate analog states. This binary switching strategy significantly reduces the complexity of the pumping system and enhances the operational repeatability. Meticulously engineered to achieve broadband achromatic focusing at a target focal length of F = 70 mm, the operational principle of this supercell in its two distinct fluidic states is conceptually illustrated in Figure 4a highlighting its reconfigurable nature under incident electromagnetic illumination.

The design process of this supercell particularly for realizing achromatic focusing when filled with LM in the ON state employed a database‐driven pick‐and‐place strategy. This strategy commenced by calculating the ideal position‐dependent phase and group delay profiles required across the supercell aperture. As depicted in Figure 4b, the target reflection phase profile in the ON state is a continuous, radially symmetric distribution meticulously engineered to ensure focusing at F = 70 mm. To achieve this achromatic focusing, each meta‐atom must provide a specific, location‐dependent phase response across the entire bandwidth. Figure 4d illustrates this by presenting the required reflection phase spectra for several representative meta‐atoms located at different radial distances *r* from the supercell's center. It is evident that while all meta‐atoms maintain a highly linear phase dispersion, their initial phase values and slopes are meticulously tailored according to their spatial position to collectively satisfy the broadband focusing requirement. Crucially, the supercell exhibits a distinctly different electromagnetic behavior in the ‘OFF’ state. As demonstrated in Figure 4c, its reflection phase profile forms a near‐equiphase surface across the aperture with minimal spatial variation. In this state, the phase response of each individual meta‐atom becomes nearly identical and non‐dispersive, regardless of its position. This is clearly shown in Figure 4e where the phase spectra for all representative meta‐atoms collapse onto a single flat line. This confirms the transition of the supercell into a non‐functional and simple reflective element when deactivated. Following the derivation of these target phase and group delay profiles for both states, the pick‐and‐place strategy involved computationally searching our extensive library of pre‐characterized meta‐atoms to find the best match for the required optical responses at each supercell location. Owing to the focusing symmetry of the supercell, a compact set of only 15 unique meta‐atoms proved sufficient to construct the entire achromatic aperture. This robust methodology effectively translates the continuous theoretical requirements for broadband achromatic focusing into a realizable and quantized physical structure.

To rigorously validate the efficacy of this design and precisely characterize the supercell's performance, comprehensive full‐wave simulations were performed, with results presented in Figure 4f,g. Both field distribution maps share the same normalized color scale for direct comparison. When the microchannels are filled with LM, the supercell acts as a highly efficient achromatic lens. As vividly illustrated in Figure 4f, the supercell consistently focuses the incident electromagnetic wave at the target focal plane (F = 70 mm) across the entire 9–14 GHz bandwidth, showing stable focusing along the X‐Z plane and clear focal spots at the focal plane. Conversely, when the microchannels are filled with NaOH solution, the focusing effect entirely vanishes, and Figure 4g clearly demonstrates that the supercell behaves as a simple planar reflector with no effective focusing. The stark contrast between these two states provides a vivid and quantitative demonstration of the supercell's function as a binary, switchable achromatic lens. To further substantiate the achromatic and broadband nature of the ‘ON’ state performance, a quantitative analysis was conducted. Figure 4h presents the cross‐sectional intensity profiles at the focal plane for various frequencies, illustrating the concentrated energy at the focal spot and its stability. Furthermore, Figure 4i plots the focal length as a function of frequency, unequivocally demonstrating that it remains consistently near the target F = 70 mm across the entire 9–14 GHz range, confirming achromatic performance. Finally, Figure 4j shows the full‐width at half‐maximum (FWHM) of the focal spot as a function of frequency, where the stable and small FWHM values indicate consistent focusing quality and resolution across the broadband operation. This high‐contrast, broadband, and achromatic switching capability is the foundational element that enables the programmable generation of complex SSP arrays.

### 2.4 Broadband Dynamic Illusion Design for Radar Detection

Following the successful validation of our reconfigurable supercells as binary, switchable achromatic lenses, our research transitioned from individual components to an integrated system: the FAM. The FAM is constructed by physically arranging 25 such supercells into a 5x5 array, collectively forming a dynamic, programmable canvas for electromagnetic illusion generation. Each supercell within this array functions as an individually addressable macropixel, whose electromagnetic state can be precisely controlled in a rapid and reversible manner.

**FIGURE 5:**
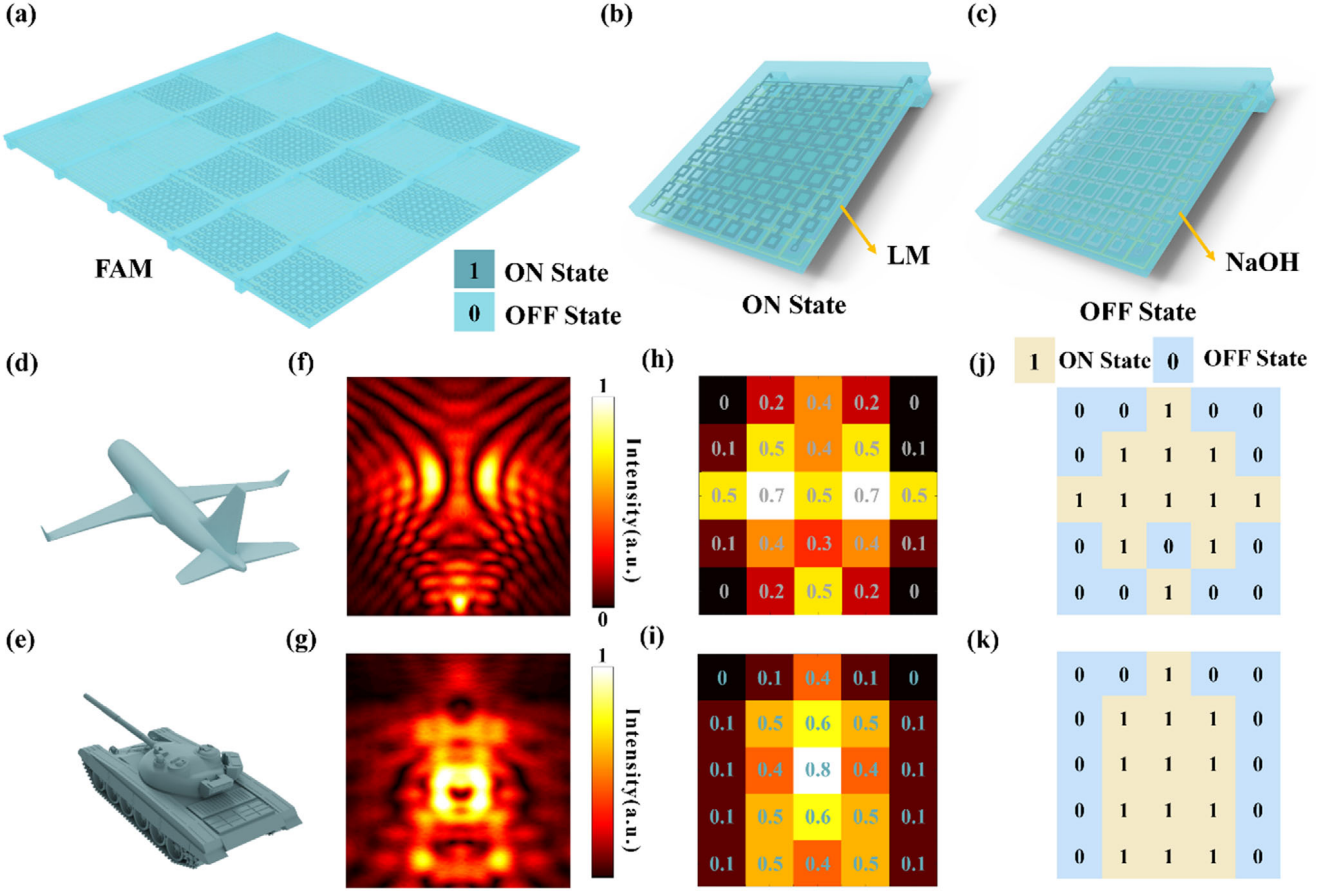
FAM design, operational principles, target characteristics, and camouflage encoding strategy. (a) Overall structural schematic of the FAM array with a coding legend. (b) Microscopic schematic of an ‘ON’ state supercell (microfluidic channels filled with LM). (c) Microscopic schematic of an ‘OFF’ state supercell (microfluidic channels filled with NaOH solution). (d, e) 3D models of aircraft, and tank targets, respectively. (f, g) Near‐field electric field distributions of the uncamouflaged aircraft, and tank targets, respectively, at a specific frequency, revealing their intrinsic radar scattering. (h, i). Average normalized near‐field electric field intensity within 25 regions for the FAM programmed to generate the signatures of an aircraft and a tank, respectively. The numbers indicate the corresponding average energy intensity values. (j, k) FAM 5x5 binary encoding maps designed for aircraft, and tank camouflage, respectively (yellow: ‘ON’, blue: ‘OFF’).

The design principles and microscopic operational mechanisms of the FAM are comprehensively presented in Figure 5. As shown in Figure 5a, a schematic illustration of the overall FAM structure is depicted, visually showcasing the array composed of multiple supercells. A clear coding legend, defining ‘1’ for the ‘ON State’ and ‘0’ for the ‘OFF State’, is also presented. The agile electromagnetic response switching capability of these supercells is achieved through the precise control of LM injection and expulsion within their microfluidic channels. Specifically, the microscopic structure of a supercell in its ‘ON State’ is shown in Figure 5b, where its microfluidic channels are filled with highly conductive LM, thereby enabling its designed electromagnetic modulation functionality. Conversely, the same supercell in its ‘OFF State’ is illustrated in Figure 5c, with its microfluidic channels filled with an electrically insulating NaOH solution, in which state the supercell primarily functions as a passive reflector. This dynamic and reversible switching between the two states forms the foundational basis for the precise control of broadband electromagnetic waves.

To systematically assess the radar illusion capabilities of the FAM, two representative military targets were selected: an aircraft and a tank, with their high‐fidelity 3D models shown in Figure 5d,e. According to high‐frequency scattering theory, the radar signature of a complex target is primarily dominated by a discrete set of SSPs which typically correspond to physical discontinuities like edges and corners. Therefore, the core task of generating a radar illusion is not to replicate the entire continuous field distribution but to strategically reconstruct the spatial arrangement of these dominant SSPs.

To extract these SSPs, we first simulated the near‐field electric field distributions of the original targets as presented in Figure 5f,g. These maps reveal the locations of high‐intensity scattering. We then employed a threshold‐based method to discretize these continuous maps and isolate the SSPs. We applied a binary encoding scheme where supercells corresponding to regions with normalized field intensity above a certain threshold are activated ('1' state) while others remain inactive ('0' state). The selection of this threshold is critical as it determines which scattering features are preserved in the final binary representation.

A comprehensive comparative analysis was performed to determine the optimal threshold value that maximizes the fidelity between the synthesized illusion and the actual target's signature. While the FAM platform can faithfully execute any encoding map, our analysis, detailed in Note S6 and Figure S5, demonstrates that an intensity threshold of 0.3 provides the best balance. This value effectively captures the primary scattering centers while filtering out noise and avoiding the over‐dilation of features that can occur with lower thresholds or the loss of secondary features associated with higher thresholds. We therefore selected 0.3 as the optimal parameter for our study. This process resulted in the binary encoding maps for each target as shown in Figure 5h,i. Although these binary maps constitute a discretized representation of the continuous near‐fields, they are explicitly designed to capture the most essential radar‐significant features. By programming the FAM to generate focused hotspots at these key locations, our approach aims to reconstruct the target's primary silhouette as it would be perceived by a high‐resolution imaging radar. The validity of this principle relies on the correct spatial synthesis of these dominant scattering points rather than a perfect replication of the entire continuous field distribution.

Consequently, these binary maps served as the precise blueprint for programming the FAM array. The state of each supercell was set to the ON or focusing state to generate a SSP at locations corresponding to a logic 1 in the map, and to the OFF or reflective state for locations corresponding to a logic 0. This strategic arrangement allows the FAM to synthesize a scattered field that mimics the signature of the desired phantom target from the perspective of the radar. The final programmed states of the FAM for the three representative targets are displayed in Figure 5j,k.

Leveraging the innovative FAM architecture and sophisticated binary encoding schemes described above, this system is capable of high‐fidelity synthesis of complex SSPs. This capability thereby effectively mimics the radar returns of diverse real‐world targets, providing a critical technological avenue for advanced radar illusion camouflage. To rigorously validate this principle and fully demonstrate the FAM's exceptional reconfigurability and broadband performance, comprehensive full‐wave electromagnetic simulations of the FAM metasurface programmed with specific SSP encodings were performed.

**FIGURE 6:**
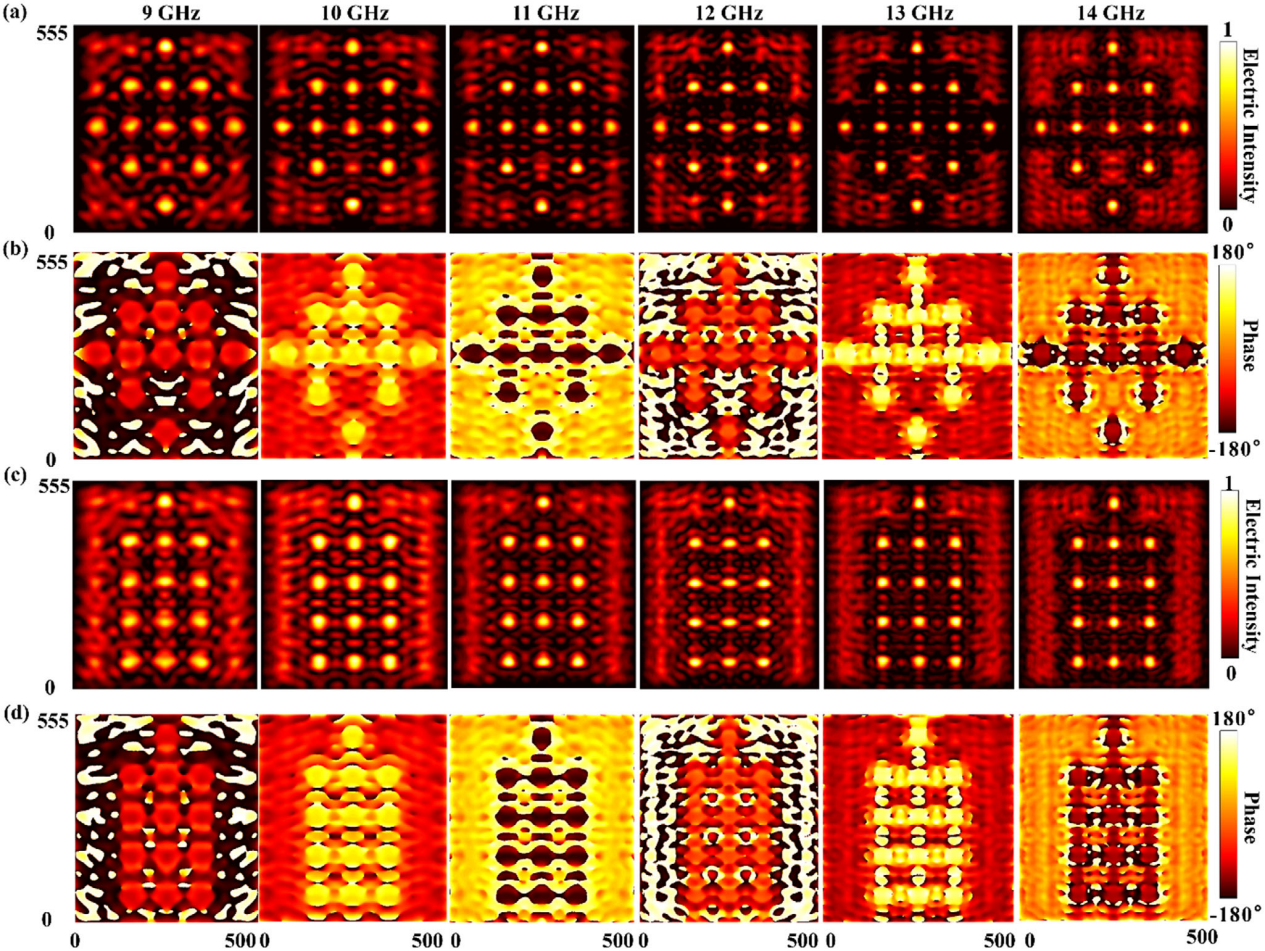
Simulated performance of the FAM for dynamic illusion camouflage. (a, c) Synthesized near‐field electric energy distributions when the FAM is programmed to mimic (a) an aircraft, and (c) a tank. (b, d) Corresponding near‐field phase profiles for the (b) aircraft, and(d) tank illusion states, illustrating the complex wavefronts generated by the FAM. All of the electric field intensities are normalized against the maximum value in the spectra.

The simulated performance of the FAM in executing dynamic illusion camouflage is comprehensively presented in Figure 6. These results validate the platform's core capability of generating tailored SSP arrays to mimic the radar signatures of phantom targets. Specifically, the synthesized near‐field electric energy distributions for the aircraft and tank illusions are shown in Figure 6a,c respectively. For each case, the FAM successfully generates a distinct SSP pattern where the high‐intensity zones spatially correspond to the desired target's key scattering centers. Complementing the energy distributions, the corresponding near‐field phase profiles are presented in Figure 6b,d. These phase maps reveal the complex and spatially varying wavefronts that are co‐generated with the SSPs which are crucial for creating a coherent and realistic far‐field scattering signature. Together, these results demonstrate that the FAM effectively orchestrates both the spatial amplitude distribution and the phase profile of the near‐field to reconstruct the required scattered field wavefront with high fidelity. This capability is not achieved through analog tuning of individual elements but rather through a spatial synthesis strategy. The amplitude distribution is defined by the selective activation of specific supercells which determines the physical locations of the high‐intensity scattering centers. Concurrently, the precise phase distribution is governed by the intrinsic achromatic geometric design of these active supercells. By strategically activating the supercells into the ON state or deactivating them into the OFF state according to the pre‐calculated encoding maps shown in Figure 5j,k, the FAM physically reconstructs the target's electromagnetic signature. This capability to physically generate complex and broadband‐stable SSP distributions represents a fundamental advancement for deceiving modern and high‐resolution radar imaging systems. In conclusion, these comprehensive simulation results provide robust validation for the FAM platform's unprecedented ability to create dynamic and convincing electromagnetic illusions.

### 2.5 Experimental Validation of the Programmable FAM

**FIGURE 7:**
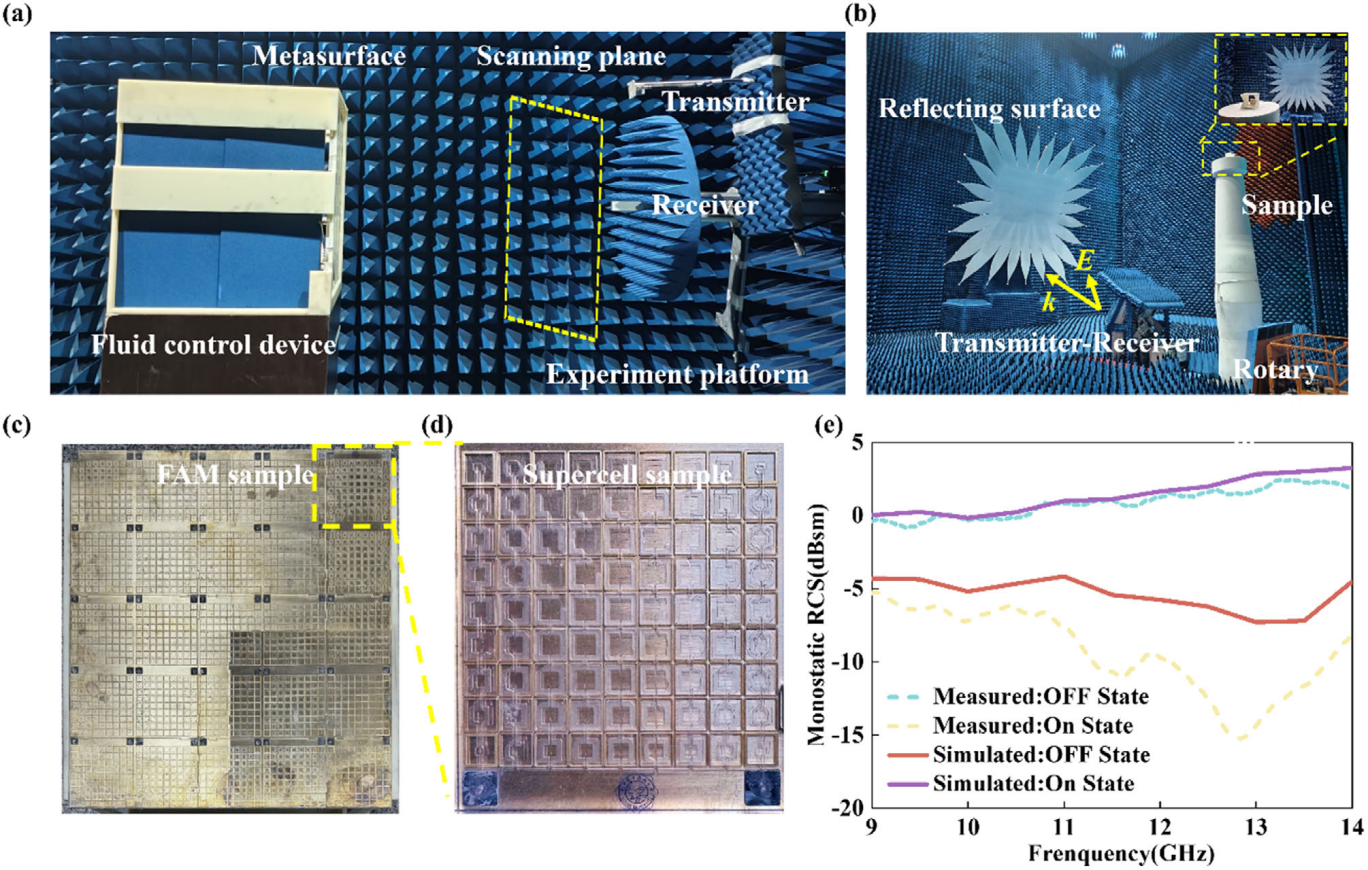
Experimental implementation and fundamental far‐field verification. (a) Photograph of the near‐field scanning measurement setup in the microwave anechoic chamber used for characterizing the full‐scale aperture. (b) Photograph of the separate far‐field measurement setup employed to characterize the scattering properties of the individual supercell. (c) The fabricated full‐scale FAM prototype (555 mm*500 mm) composed of spliced PCB panels. (d) Close‐up view of the representative supercell. (e) Far‐field RCS validation of the supercell. The measured (solid lines) and simulated (dashed lines) far‐field scattering patterns of the supercell.

To provide definitive experimental validation for the proposed FAM paradigm, a full‐scale prototype was fabricated and systematically characterized using both near‐field and far‐field measurement techniques. The comprehensive experimental implementations are presented in Figure 7.

The measurements were conducted in a microwave anechoic chamber. The experimental setup for near‐field scanning, which was employed to characterize the large‐scale aperture field distribution, is illustrated in Figure 7a. Complementarily, to rigorously evaluate the scattering properties in the far‐field, a separate far‐field measurement setup was established, as depicted in Figure 7b. The physical realization of the large‐area (555 mm*500 mm) FAM prototype is presented in Figure 7c, which was assembled from multiple smaller PCB panels. A detailed view of a representative supercell is shown in Figure 7d (Full fabrication and setup details are provided in the Experimental Section).

Validating the electromagnetic camouflage performance requires establishing consistency between the fundamental scattering supercell and the macroscopic array. Before evaluating the complex illusions, the far‐field modulation capability of the basic building block was verified using the setup shown in Figure 7b. The measured far‐field scattering patterns of the supercell are presented in Figure 7e. The measurement results, represented by solid lines, show excellent agreement with simulations, represented by dashed lines. Specifically, the supercell exhibits a notably higher scattering intensity when filled with NaOH solution compared to the liquid‐metal‐filled state. The two states display distinct scattering characteristics, thereby experimentally confirming the robust capability of the proposed structure to modulate far‐field scattering. This high‐contrast switching lays the physical foundation for the array‐level camouflage.

For the full‐scale FAM array, direct far‐field measurement of the illusionary targets (aircraft and tank) is constrained by the physical aperture size, which would require a test distance exceeding 40m ($R>\frac{2D^{2}}{\lambda }$) at X‐band. Therefore, the high‐precision near‐field scanning shown in Figure 7a was relied upon to map the aperture field distribution, which physically determines the far‐field pattern via the Huygens‐Fresnel principle.

A baseline measurement of the prototype in its uniform “all‐OFF” state revealed systematic background field variations across the aperture, as discussed in Note S8. These variations are attributed to the incident field tapering and minor planar deviations at the panel seams due to the vertical mounting required for scanning. Crucially, despite these systemic non‐idealities, the programmed electromagnetic contrast of the FAM proved sufficiently robust. Although a direct vectorial background subtraction was constrained by experimental logistics, the signal‐to‐noise ratio was high enough to resolve the target topology. The primary objective was to verify the spatial accuracy of the SSPs, ensuring that the relative field intensity peaks align with the intended camouflage pattern.

The practical viability of the fluidic system was rigorously assessed. The prototype underwent over 50 complete reconfiguration cycles with no leakage or degradation, as visualized in Video S1. While the fluidic switching speed (∼45s) is inherently different from nanosecond‐scale electrical switches, the FAM offers superior dispersion engineering capabilities that electrical counterparts lack. As detailed in the quantitative comparison in Note S1, unlike PIN diodes that are typically limited to fixed phase states, the FAM enables simultaneous control of phase and group delay through geometric reconfiguration, which is essential for the demonstrated broadband achromaticity. Furthermore, its all‐metallic structure offers ultra‐high power handling capabilities theoretically limited only by air breakdown, far exceeding the thermal limits of semiconductor junctions, as detailed in Note S2.

**FIGURE 8:**
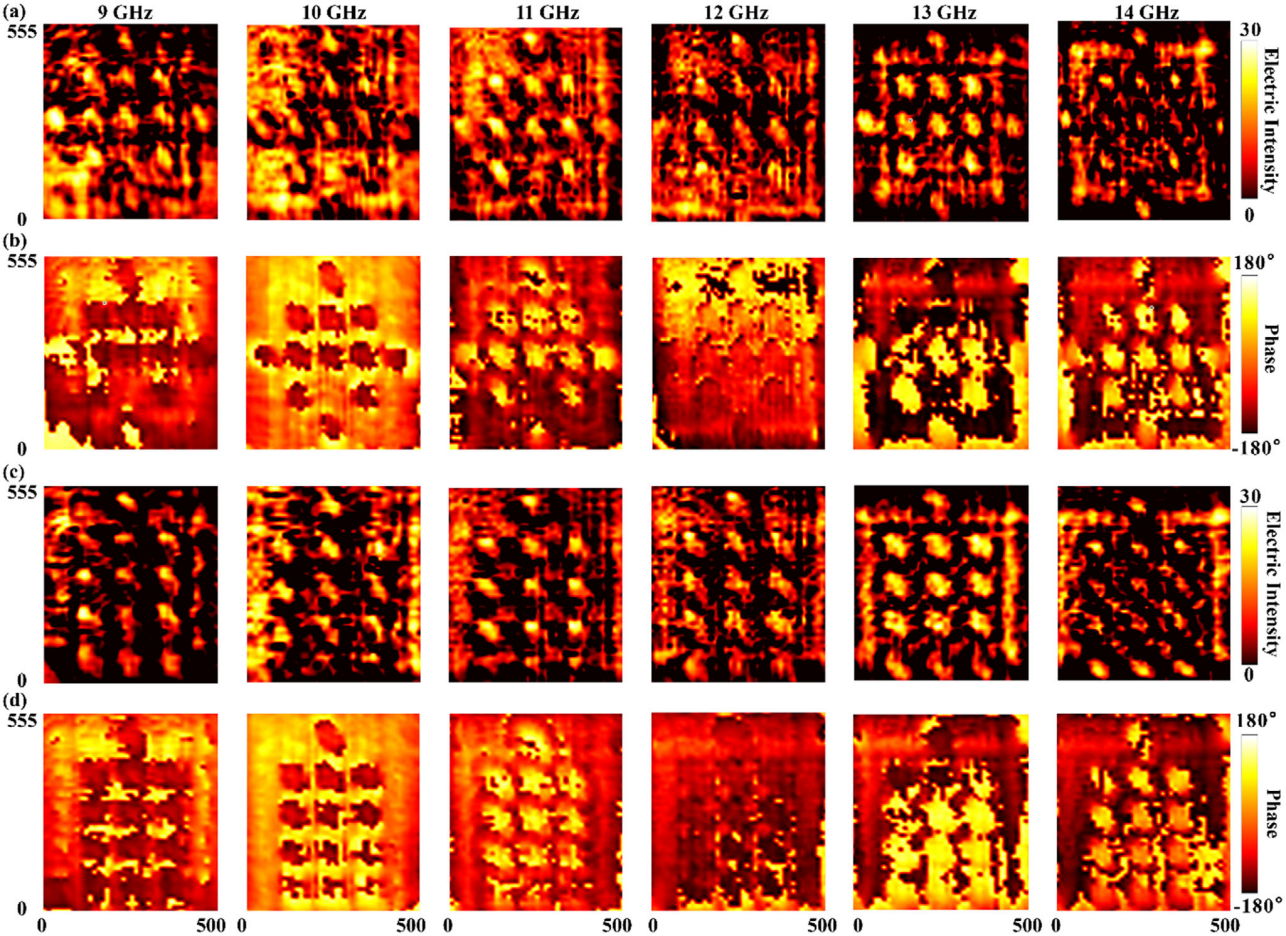
Experimental realization of broadband illusion camouflage. (a, c) Measured normalized near‐field electric energy distributions for the aircraft (a) and tank (c). (b, d) Measured phase distributions for the aircraft (b) and tank (d), showing the complex wavefront reconstruction co‐generated with the strong scattering points.

With the reliability established, the FAM was programmed to generate scattering signatures for an aircraft and a tank. The experimental results are summarized in Figure 8. The measured near‐field electric energy distributions for the aircraft and tank are displayed in Figure 8a,c, respectively. These maps reveal sharply defined SSP contours. Critically, the high‐energy zones are correctly synthesized at the programmed locations, such as the fuselage/wings and chassis/turret, demonstrating strong structural agreement with the simulation results despite the background modulation. Complementing these energy maps, the corresponding phase profiles, presented in Figure 8b,d, exhibit the complex, spatially varying wavefronts that are co‐generated with the SSPs. The successful reconstruction of these intricate amplitude and phase distributions on the aperture experimentally validates the FAM's capability to synthesize the electromagnetic fields required for broadband illusion camouflage.

## 3 Conclusions

In summary, we have developed and experimentally validated a novel paradigm for dynamic, broadband electromagnetic illusion based on a FAM. Our approach, which leverages the programmable synthesis of achromatic SSPs, successfully overcomes the bandwidth and reconfigurability limitations inherent in conventional camouflage metasurfaces. The cornerstone of our platform is a quasi‐3D, multi‐resonant meta‐atom that provides unprecedented control over phase dispersion. By assembling these meta‐atoms into switchable, achromatic focusing supercells, we constructed a fully programmable array capable of generating high‐fidelity SSP patterns that mimic various targets. The experimental validation, demonstrating near‐perfect agreement with simulations across a wide 9‐14 GHz bandwidth, confirms the robustness of our design methodology and the reliability of the fluidic actuation system. This work transitions the concept of illusion camouflage from static, narrowband devices to a dynamic, intelligent, and broadband system. The demonstrated capabilities, which include real‐time switching between complex radar signatures like aircraft, drones, and tanks, establish a viable and scalable pathway toward next‐generation adaptive stealth technologies. We believe the principles and platform developed herein will lay a crucial foundation for future advancements in military defense, smart sensors, and secure wireless communications.

## 4 Experimental Section

### 4.1 Electromagnetic Simulation Setup

The meta‐atom simulations and full‐wave analysis are conducted using CST Microwave Studio. Periodic boundary conditions are applied along the y‐axis, while radiation boundary conditions are set along the x‐ and z‐axes. In the full‐wave simulations, electric field monitors are employed to examine the field distributions in the x‐y and y‐z planes.

### 4.2 Sample Fabrication

Based on the design specifications, the corresponding PMMA dielectric substrate was prepared, and the substrate was cut using a high‐speed machine. Copper‐based metal square rings were also fabricated. All samples were then washed with purified water. The dimensions of the samples were measured and compared to the required specifications. Upon meeting the dimensional requirements, the samples were baked at 55°C, after which they were cleaned again. The metal square rings were placed into the corresponding recesses on the substrate. Subsequently, all substrates were thermally bonded at 75°C to obtain the final independent samples.

### 4.3 Electromagnetic Experimental Setup

All electromagnetic measurements were performed within a microwave anechoic chamber to completely eliminate environmental clutter and interference. The experimental characterization was divided into two distinct configurations to evaluate both the macroscopic aperture fields and the unit‐level scattering properties.

### 4.4 Near‐Field Scanning Setup

For the full‐scale FAM prototype, a high‐precision near‐field scanning system was employed. To ensure near‐plane‐wave illumination, two standard gain horn antennas were used as the transmitting feeds. These horns were fed by two distinct waveguide‐to‐coaxial adapters, operating over the 8.2–12.4 GHz and 9.84–15 GHz bands respectively, ensuring full coverage of the operational bandwidth. As illustrated in Figure 7a, the FAM prototype was positioned vertically in front of the feed horn. An electric field probe was utilized to precisely map the planar field distribution at a distance of 70 mm from the FAM's surface. During the measurement process, the probe scanned a planar area of 555 mm × 500 mm, while its relative position to the FAM was kept constant.

A key challenge in characterizing reflective metasurfaces is that the feed and the probe must reside on the same side of the device under test. This configuration, coupled with the probe's restricted movement over a metallic scanning plane, inherently risks physical blockage and near‐field coupling between the incident horn and the probe. To mitigate the influence of the test apparatus on the results, the incident wave was intentionally set at a small angle of incidence to illuminate the metasurface, thereby maximizing the accuracy and reliability of the collected near‐field data.

### 4.5 Far‐Field Measurement Setup

To rigorously validate the scattering modulation capability of the fundamental building block, a separate far‐field measurement configuration was established, as shown in Figure 7b. The representative supercell was mounted on a low‐scattering foam support to minimize background noise. Transmitting and receiving horn antennas were positioned at a distance satisfying the far‐field criterion ($R>\frac{2D^{2}}{\lambda }$) relative to the supercell dimension. A Vector Network Analyzer was connected to the antennas to record the scattering parameters. This setup allowed for the direct comparison of the RCS characteristics between the liquid‐metal‐filled and NaOH‐filled states, providing the physical ground truth for the simulation results.

### 4.6 Fluid Experimental Setup

The reconfiguration of the metasurface is achieved through a precise pressure‐driven microfluidic system. To ensure stable and reversible actuation, we employed a binary fluid strategy consisting of a finite volume of LM acting as a conductive slug encapsulated by a NaOH solution. The NaOH solution serves three critical roles in this system. First, it acts as a hydraulic transmission medium to transfer pressure from the external syringes to the LM. Second, it functions as an effective anti‐oxidation agent that prevents the formation of oxide skin on the LM surface ensuring residue‐free motion within the channels. Third, it serves as the dielectric filling for the OFF state to minimize impedance mismatch. The switching mechanism operates between two distinct physical states. In State 0 or the Dielectric State, the resonant cavity of the meta‐atom is filled entirely with the NaOH solution which behaves as a dielectric substrate with low permittivity contrast. In State 1 or the Metallic State, the LM slug is injected into the active region to displace the NaOH solution thereby establishing a strong metallic resonance. The actuation is controlled by regulating the pneumatic pressure at the channel inlets and outlets using high‐precision syringes. This setup ensures that the LM slug is always strictly confined between columns of NaOH solution to maintain high repeatability over multiple reconfiguration cycles.

## Conflicts of Interest

The authors declare no conflicts of interest.

## Supporting information




**Supporting File 1**: advs73745‐sup‐0001‐SuppMat.docx.

**Supporting File 2**: advs73745‐sup‐0002‐Video S1.mp4.

## Data Availability

The data that support the findings of this study are available from the corresponding author upon reasonable request.
