# Supplementary material for: Broadband Achromatic Programmable Electromagnetic Camouflage via Fluidic‐Accessible Metasurface
Source: Adv Sci (Weinh). 2026 Jan 9;13(10):e23971. doi: 10.1002/advs.202523971 (PMC12915077; doi:10.1002/advs.202523971)
Supplement: Supplementary file 1 — Supporting File 1: advs73745‐sup‐0001‐SuppMat.docx. [file ADVS-13-e23971-s001.docx]

Supporting Information

Broadband Achromatic Programmable Electromagnetic Camouflage via Fluidic-Accessible Metasurface

Shipeng Liu^1^, Jiahao Zhang^1^, Jiale Wang^1^, Zeyao Chen^1^, Zhaotong Yu^1^, Jinhong Luo^1^, Jiabao Luo^1^, Shuxi Gong^1^, Qingxin Guo^3^, Ping Li^4^, Rujiang Li^1^, Ke Chen^2^, Yongtao Jia^1^, Ying Liu^1^,

Supplementary Information

Broadband Achromatic Programmable Electromagnetic Camouflage via Fluidic-Accessible Metasurface

Shipeng Liu^1^, Jiahao Zhang^1^, Jiale Wang^1^, Zeyao Chen^1^, Zhaotong Yu^1^, Jinhong Luo^1^, Jiabao Luo^1^, Shuxi Gong^1^, Qingxin Guo^3^, Ping Li^4^, Rujiang Li^1^, Ke Chen^2^, Yongtao Jia^1^, Ying Liu^1^,

^1^School of Electronic Engineering, Xidian University, Xian 710071, China.

^2^School of Electronic Science and Engineering, Nanjing University, Nanjing 210093, China

^3^School of Information and communication Engineering, Communication University of China, Beijing 100024, China.

^4^Applied Physics Technology Center, Beijing Institute of Mechanical Equipment, Beijing 100854, China.

The supplementary Information includes:

Supplementary Note 1: Quantitative Comparison with Existing Dynamic Metasurfaces

Supplementary Note 2: Power Capacity Analysis of Liquid Metal-based FAM

Supplementary Note 3: Design Principles for Meta-Atoms in FAM

Supplementary Note 4: Database construction for Meta-Atoms in FAM

Supplementary Note 5: Detailed design of the FAM

Supplementary Note 6：Far-Field Performance Validation and Theoretical Consistency

Supplementary Note 7: Optimization and Validation of Encoding Threshold Parameters

Supplementary Note 8: Characterization of Background Field Non-uniformity in the Large-Area FAM Prototype

Supplementary Note 9: Microfluidic Design, Infusion Protocol, and Stability Analysis

Other Supplementary Materials for this manuscript include the following:

Supplementary Video S1 (.mp4 format). Reconfiguration process of a supercell via fluidic infusion.

Supplementary Note 1: Quantitative Comparison with Existing Dynamic Metasurfaces

To comprehensively evaluate the performance of the proposed Fluidic-Accessible Metasurface (FAM), we benchmarked it against state-of-the-art reconfigurable metasurface technologies, including PIN diodes, Micro-Electro-Mechanical Systems (MEMS), and Vanadium Dioxide (VO₂) phase-change materials. Table S1 summarizes the key performance indicators, focusing on bandwidth, dispersion engineering capability, insertion loss, power consumption, and power handling capacity.

**Supplementary Table S1:** Quantitative Comparison between the proposed FAM and existing dynamic metasurface technologies.

| **Indicators** | **This Work (FAM)** | **PIN Diode Metasurfaces** | **MEMS Metasurfaces** | **VO₂ Metasurfaces** |
| --- | --- | --- | --- | --- |
| **Dispersion Engineering** | Arbitrary control of phase and group delay | Limited | Limited | Limited |
| **Group delay control bandwidth** | Broadband (~44.4%, 9–14 GHz) | Narrow（about 3%） | Narrow | Narrow |
| **Response Speed** | Slow (~45 s for supercell injection) | **Very Fast (ns)** | Fast (µs) | Fast (ns–µs) |
| **Insertion Loss** | Low (< 0.5 dB) | Moderate | Low | High |
| **Static Power Consumption** | Zero (Pressure-balanced latching state) | High | Zero or Low | High |
| **Power Handling** | High (Solid-liquid metal hybrid, no junction breakdown) | Low/Medium | Medium | Medium |
| **Complexity** | Low (Microfluidic channels) | High | High | Moderate |
| **Cycle Life** | Moderate (>50 verified) | Very High | High | High |
| **Cost** | Low | Moderate | Very High | High |

Based on the data presented in the table, we would like to highlight three critical advantages of the FAM that distinguish it from conventional electrically controlled counterparts:

**1. Superior Dispersion Engineering Capability**

Based on the quantitative comparison, the FAM exhibits a fundamental advantage over conventional electrically controlled counterparts in terms of dispersion engineering. Existing metasurfaces based on PIN diodes or MEMS switches are typically limited to discrete switching between fixed phase states (e.g., 0 and $\pi$) with constrained dispersion profiles. They often lack the capability to independently manipulate the group delay ($\tau=-\frac{df\left( \omega\right)}{\omega}$), leading to intrinsic difficulties in eliminating chromatic aberration across a broad spectrum.

In contrast, our FAM leverages the continuous physical reconfiguration of the liquid metal (LM) geometry to precisely tune the resonant modes. This mechanism enables the simultaneous and flexible regulation of both phase response and group delay. This capability is pivotal for realizing true achromaticity and high-fidelity illusion camouflage over an ultra-wide bandwidth, a feat that is challenging for simple phase-switching devices to achieve.

**2. Zero Static Power and High Power Handling**

The FAM offers distinct advantages in energy efficiency and power resilience through its unique latching mechanism and all-metallic construction.

**2.1 Zero Static Power**

The system utilizes a pressure-balanced microfluidic latching mechanism. Once the LM is injected into the target channel, the internal pressure equalizes and maintains the state without any external energy input. This contrasts sharply with PIN diodes, which require continuous bias current to maintain the ON state and lead to substantial power drain in large-scale arrays.

**2.2 High Power Handling Comparison**

We conducted a rigorous calculation of the power capacity to demonstrate the superiority of our device. The power handling of the FAM is limited only by the breakdown field strength of the environment rather than the thermal burnout of semiconductor junctions.

We determined the breakdown threshold based on the Kilpatrick Criterion. For a device operating in air at room temperature and atmospheric pressure, the limiting factor is the breakdown field strength of air which is approximately 3 MV/m based on uniform electric field discharge experiments. For vacuum environments, this threshold increases to the dielectric breakdown limit of 4 MV/m. Using the formula: , where *E*ₘₐₓ denotes the simulated maximum electric field, ***Pest*** denotes the power capacity, *DS* denotes the breakdown field strength, and ***Pin*** denotes the input power, which is typically set to 1 W. We calculated that the maximum power capacity of our FAM reaches approximately 1.2 * 10^10 W. We have provided the detailed derivation process and calculation steps in Note **S2**(Supporting Information).

In contrast, commercial diodes are severely limited by their thermal power handling capacities. According to standard datasheets, varactors such as the SMV1233-004LF and MV1235-011 have power ratings of only 250 mW and 0.25 W respectively. Even high-power PIN diodes like the MADP-011029 and MASW-010647 are limited to approximately 7.5 W and 20 W. Even if we consider a theoretical parallel connection of these diodes, the aggregate power capacity remains significantly lower than that of the FAM. This analysis confirms that our fluidic approach is uniquely robust against high-power microwave interrogation.

**3. Low Insertion Loss**

By eliminating the parasitic resistance and capacitance associated with semiconductor diodes and their complex feed networks, the FAM achieves an exceptionally low insertion loss of less than 0.5 dB. This ensures maximum scattering efficiency and enhances the stealth performance of the generated illusions.

**4. Operational Sustainability: Cost and Cycle Life Analysis**

**4.1Cycle Life**

While we acknowledge that the switching speed and cycle life of the microfluidic system are lower than those of solid-state switches, the FAM is positioned as a quasi-static reconfiguration platform. The verified cycle life of over 50 cycles is sufficient for its intended applications such as pre-mission camouflage configuration where stability and power autonomy are paramount.

**4.2 Cost Efficiency Analysis (500 mm × 500 mm Aperture)**

A comparative cost analysis for a large-aperture metasurface (500mm*500mm) reveals the significant economic advantage of the FAM in high-power scenarios.

The FAM is constructed primarily from a commercial-grade PMMA dielectric substrate with dimensions of 500mm*500mm*6mm and utilizes a binary fluid system. Based on the fluidic channel design where a single supercell has a volume of 0.81 mL, the total weight of the LM required to fill the entire structure is approximately 125 g. This corresponds to a material cost of roughly $140 for the LM. Although the initial fabrication of a high-precision industrial mold for a panel of this size incurs a substantial one-time Non-Recurring Engineering (NRE) cost estimated at approximately $7,000, the subsequent marginal production cost per unit is extremely low due to the use of inexpensive polymer injection molding and bulk fluid materials.

In contrast, constructing a PIN diode-based metasurface of the same dimensions requires a high-frequency dielectric laminate such as Rogers 4003C or 5880 which significantly increases the substrate cost to approximately $500 per panel. Furthermore, assuming a half-wavelength periodicity, the array requires approximately 2,500 discrete unit cells. To match the kilowatt-level power handling capacity of the FAM, high-power PIN diodes are essential. With a typical market price of $5 to $10 per high-power diode, the component cost alone ranges from $12,500 to $25,000. This excludes the substantial additional costs associated with complex soldering and biasing network integration. Therefore, the FAM offers a highly scalable and cost-effective solution for large-area smart skins where the cost per unit area is driven primarily by the initial tooling rather than the exponential scaling of expensive electronic components.

Regarding the response speed, we acknowledge that the fluid injection process for a full supercell takes approximately 45 seconds. Therefore, we have clarified in the revised manuscript that the current FAM system is positioned as a quasi-static reconfiguration platform. It is ideally suited for applications such as pre-mission camouflage configuration for ground vehicles like tanks or stationary high-value targets, where the priority is placed on broadband stealth quality, power autonomy, and survivability rather than nanosecond-level switching.

Supplementary Note 2: Power Capacity Analysis of LM-based FAM

In this section, we analyze the power capacity of the FAM and compare it with traditional diode-based systems. The proposed FAM, utilizing LM and solid metal structures, avoids the low breakdown thresholds associated with semiconductor junctions.

The calculation method for the FAM power capacity is established as follows:

a) Determining the voltage value corresponding to the breakdown field strength:

b) The value of the breakdown field strength in a vacuum can be determined based on the Kilpatrick Criterion:

$f\left(MHz\right)=1.643E_{\max}^{2}e^{\frac{-8.5}{E_{\max}}}$ Eq. S1

As shown in the **Figure S1**, at a frequency of 10 GHz, the breakdown field strength of a vacuum is 69.7 MV/m.


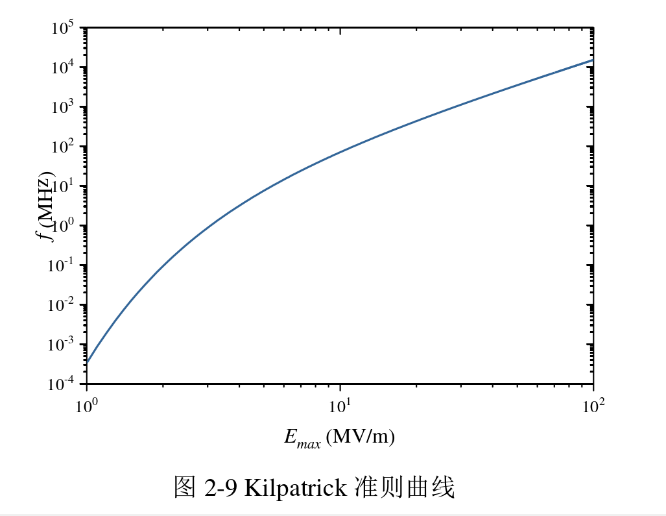


**Figure S1**. Kilpatrick Criterion Curve

c) The electric field distribution is simulated with an input power of 1 W, and the maximum electric field value (Eₘₐₓ) is extracted.

d) The antenna power capacity is calculated using the formula:

Eq. S2

Where ***P_est_*** denotes the power capacity, ***DS*** denotes the breakdown field strength, and ***P_in_*** denotes the input power, which is typically set to 1 W.

The minimum value of the breakdown field strength among antenna components is selected to calculate the power capacity of the device. In terms of breakdown field strength, the order is as follows: vacuum (metal) breakdown > vacuum (solid dielectric) breakdown > vacuum (liquid dielectric) breakdown > inert gas breakdown > air breakdown.

First, the operating environment of the device must be clarified. If the FAM operates in a vacuum environment, the minimum breakdown field strength is determined by the dielectric substrate, and 4 MV/m is selected as the breakdown field strength. If it operates in air at room temperature and atmospheric pressure, the minimum breakdown field strength is that of air, and 3 MV/m is selected.

To accurately evaluate the breakdown threshold, the electric field distribution is simulated based on a supercell of the FAM. Due to the periodic nature of the metasurface structure, the electromagnetic response within the supercell is representative of the entire array. With an input power of 1 W applied to the supercell, the maximum electric field value on the surface is extracted as 27.6 V/m.


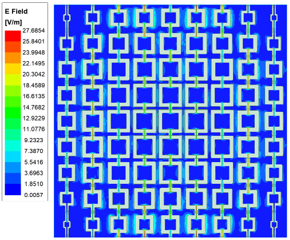


**Figure S2**. **Electric field distribution on the FAM supercell surface.**

When considering the FAM operating in air at room temperature and atmospheric pressure, the breakdown field strength is equal to that of air (3 MV/m). Based on the aforementioned formula, the power capacity can be obtained.

$$P_{est}=\left(\frac{3\times10^{6}V/\text{m}}{27.6V/\text{m}}\right)^{2}\times1W=1.18\times10^{10}W$$

In a vacuum environment, the breakdown field strength of the FAM is limited by the dielectric plate (4 MV/m). Consequently, the power capacity is calculated accordingly using the scaling formula derived above.

$$P_{est}=\left(\frac{4\times10^{6}V/\text{m}}{27.6V/\text{m}}\right)^{2}\times1W=2.10\times10^{10}W$$

Meanwhile, according to the varactor diode datasheets, varactors typically have relatively low power handling capacities. Taking the varactor and PIN diodes as an example: the power capacity of MV1235-011 is 0.25 W; that of SMV1233-004LF is 250 mW; that of MADP-011029-14150T is 7.5 W; that of MASW-010647 is 20 W; the power capacity of MA4P4001BNM-402 is less than 12 W; and that of MADP-011028-14150T is 3.3 W.

Even when high-power PIN diodes are utilized, the maximum power capacity of a diode-based array remains orders of magnitude lower than the breakdown-limited capacity of the FAM. This analysis confirms that the proposed FAM is exceptionally suitable for high-power radar and dynamic illusion applications where traditional semiconductor-based metasurfaces may fail.

Supplementary Note 3: Design Principles for Meta-Atoms in FAM

As discussed in the main text, the key to realizing broadband and reconfigurable achromatic focusing lies in the design of meta-atoms with tunable phase and phase dispersion. To this end, we employ multilayer quasi-3D meta-atoms, in which the resonance frequencies and strengths of both intra-layer self-resonances and inter-layer mutual resonances are judiciously engineered. As illustrated in **Figure 2a**, each meta-atom consists of a microfluidic layer, a solid metallic layer, and a metallic ground plane separated by PMMA spacers. The microfluidic channel can be filled with either LM or a NaOH solution, thereby enabling reconfigurability.

According to the equivalence principle of electromagnetics, when a TE-polarized wave impinges on a meta-atom with LM in the microchannel, opposite surface currents are induced at the upper and lower interfaces of the microchannel and the metallic layer, as shown in **Figure S3.** These antiparallel currents generate Lorentz-type resonances, which can be described by the classical Lorentz oscillator model:^[1]^

$\left( \omega_{n}^{2}-\omega^{2}-i\gamma_{n}\omega\right)m_{n}\left( \omega\right)=f_{n}(\omega)H_{loc,n}(\omega)$ Eq. S3

where $\omega_{n}$, $\gamma_{n}$，$m_{n}\left( \omega\right)$ and $f_{n}\left( \omega\right)$ denote the resonance frequency, damping, magnetic dipole moment, and coupling coefficient of the $n^{th}$ layer, respectively. The local magnetic field is given by the sum of the incident field and the scattered fields from other layers:

$H_{loc,n}=H_{inc}+\sum_{m\neq n} g_{mn}m_{m}(\omega)$ Eq. S4

with $g_{mn}$​ representing the inter-layer coupling coefficient. Substituting Eq. S2 into Eq. S1 yields:

$\left( \omega_{n}^{2}-\omega^{2}-i\gamma_{n}\omega\right)m_{n}\left( \omega\right)-\sum_{m\neq n} f_{n}g_{mn}m_{m}(\omega)=f_{n}(\omega)H_{inc}$ Eq. S5

Thus, the overall response of the system can be expressed as a weighted summation of the magnetic dipole moments:

Eq. S6

where is the effective magnetic polarizability and $c_{n}$​ represents the observation weight of each dipole. The coupled system can be expanded into a series of Lorentz resonances, approximated as:

Eq. S7

leading to the overall reflection phase of the meta-atom:

$\varphi\left( \omega\right)=arg\left[ \sum_{n=1}^{N} \frac{A_{n}e^{i\theta_{n}}}{\omega^{2}-\omega_{n}^{2}+i\gamma_{n}\omega} \right]$ Eq. S8

This analytical framework clearly demonstrates that by precisely tailoring both the intra-layer and inter-layer coupling strengths, as well as their relative contributions, the reflection phase profile and phase dispersion can be systematically engineered, thereby enabling broadband achromatic focusing. As shown in Figures S3a–c, when different types of magnetic resonances are incorporated within the designed meta-atom, the reflection amplitude remains above –0.3 dB, while the reflection phase exhibits an almost perfectly linear response—satisfying the targeted electromagnetic performance requirements. Figure S3d further plots the phase distributions corresponding to various resonance types and mode numbers, indicating that modifying the resonance type effectively extends the coverage range of the linear phase dispersion. Moreover, when the microchannel is filled with a NaOH solution, the magnetic resonance in the fluidic layer is suppressed, enabling dynamic switching between the achromatic focusing state and the planar reflection state.


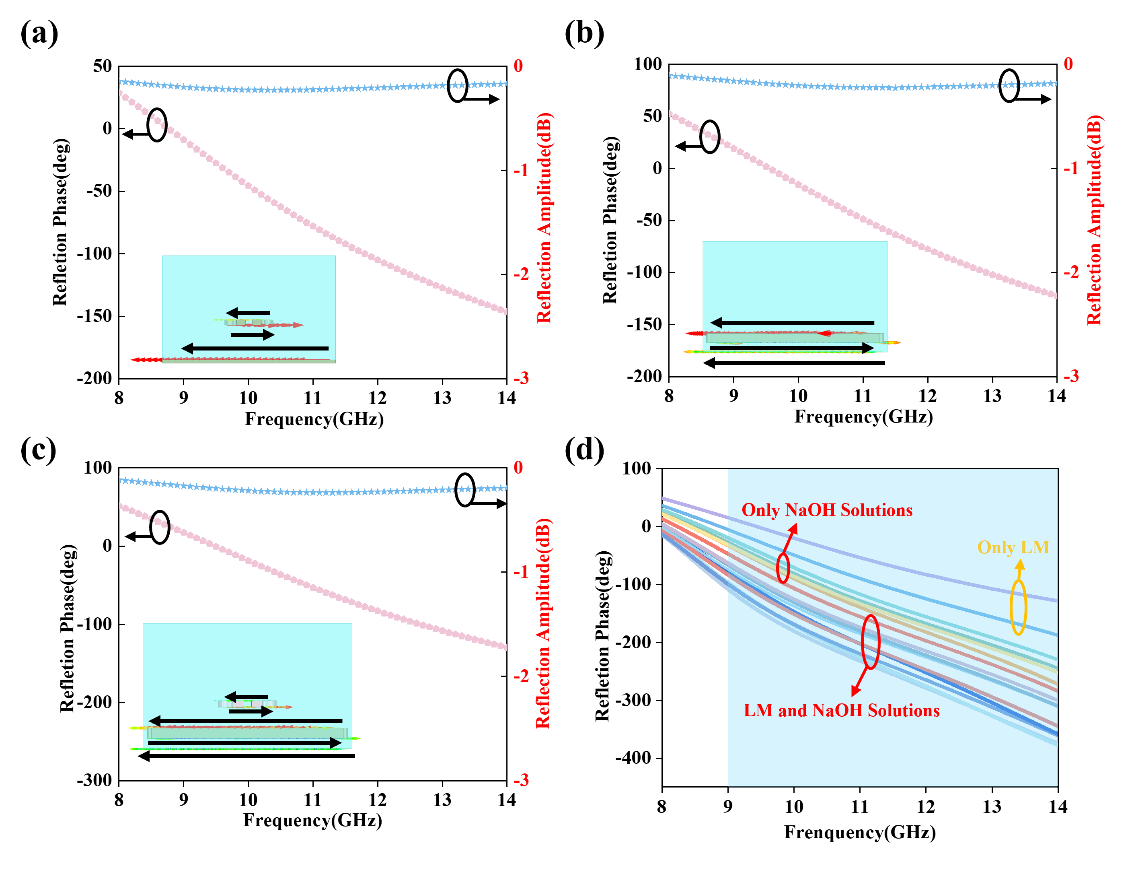


Figure S3. Engineered linear phase dispersion for broadband achromatic metasurfaces. a, b, c) Reflection amplitude and phase profiles for meta-atoms featuring different magnetic resonance types. The reflection amplitude is maintained above –0.3 dB, ensuring high efficiency. d) Achievable phase distributions for various resonance types and mode numbers, demonstrating that tailoring the resonance type systematically extends the linear phase coverage. This versatile phase control, combined with the ability to suppress the magnetic resonance by filling the microchannel with a NaOH solution, provides the fundamental mechanism for switching between the achromatic focusing state and a planar reflection state.

Supplementary Note 4：Database construction for Meta-Atoms in FAM

we constructed the reflection characteristic database of the designed meta-atom. Due to the actual processing limitations of the microfluidic chip, the remaining parameters (a, b) also have certain limitations. Based on the above characteristics, a database of meta-atom phase and phase slope is established.

The changes of each parameter are listed below:

**Supplementary Table S2.** The size database of element meta-atoms with different reflection coefficients

| Parameters | Starting point | Step | End point |
| --- | --- | --- | --- |
| *a_1_* | 9.8mm | 0.1mm | 2mm |
| *b_1_* | 9.2mm | 0.1mm | 1mm |
| *a_2_* | 9.8mm | 0.1mm | 2mm |
| *b_2_* | 9.2mm | 0.1mm | 1mm |

We simulated the meta-atoms with different structural parameters by CST Microwavestudio, and obtained the amplitude and phase response at 9-14 GHz. At the same time, the selection process is implemented for all meta-atoms based on the target of low loss and linear phase distribution. Here, we first determine its average amplitude $\chi$, and its $\chi_{\mathrm{target}}$ is set to 0.9. If the average amplitude of the meta-atom is less than $\chi_{\mathrm{target}}$, the element atom is not recorded. If the average amplitude meets the requirements, the phase distribution of the meta-atom in the working band is fitted by a linear fitting function. According to the obtained fitting coefficient and root mean square error, the linear phase shift and accuracy are explained, and the meta-atom that meets the requirements is finally recorded.

**Supplementary Note 5：****Detailed design of the FAM**

According to **Figure 3b** in the main text, when the phase profile at different heights changes with frequency, our meta-atoms should show the corresponding linear reflection phase spectrum in a broadband range. For this reason, according to the meta-atom database in **Supplementary Note 4**, fifteen meta-atoms that are closest to the required linear phase spectrum are selected for further optimization to make them more consistent with the required phase. **Supplementary Table S3** shows the detailed atomic parameters of the FAM. **Figure S4** shows the reflection amplitude and reflection phase distribution of the designed fifteen meta-atoms filled with LM and NaOH solution respectively. We note that the reflection amplitude of each atom of the designed cloak is greater than 0.95 in the operating frequency band of 9-14 GHz, achieving extremely low loss.

**Supplementary Table S3.** The meta-atom structure size of the quasi-three-dimensional microfluidic dynamic camouflage cloak

| NO. meta-atom | 1 | 2 | 3 | 4 | 5 | 6 | 7 | 8 | 9 | 10 | 11 | 12 | 13 | 14 | 15 |
| --- | --- | --- | --- | --- | --- | --- | --- | --- | --- | --- | --- | --- | --- | --- | --- |
| a_1_ | 9.2 | 9.1 | 9 | 8.8 | 8.7 | 8.4 | 8.3 | 8.1 | 7.7 | 6.9 | 7.2 | 7.1 | 6.5 | 5.4 | 2.8 |
| b_1_ | 6.3 | 6.2 | 6.1 | 5.9 | 5.7 | 5.3 | 5 | 5.1 | 4.7 | 4 | 4.2 | 4.1 | 4.8 | 3.4 | 1.8 |
| a_2_ | 9.6 | 9.6 | 9.6 | 9.6 | 9.6 | 9.6 | 9.6 | 9.6 | 9.6 | 9.6 | 9.6 | 9.6 | 9.6 | 9.6 | 9.6 |
| b_2_ | 8.8 | 8.8 | 8.8 | 8.8 | 8.8 | 8.8 | 8.8 | 8.8 | 8.8 | 8.8 | 8.8 | 8.8 | 8.8 | 8.8 | 8 |

**
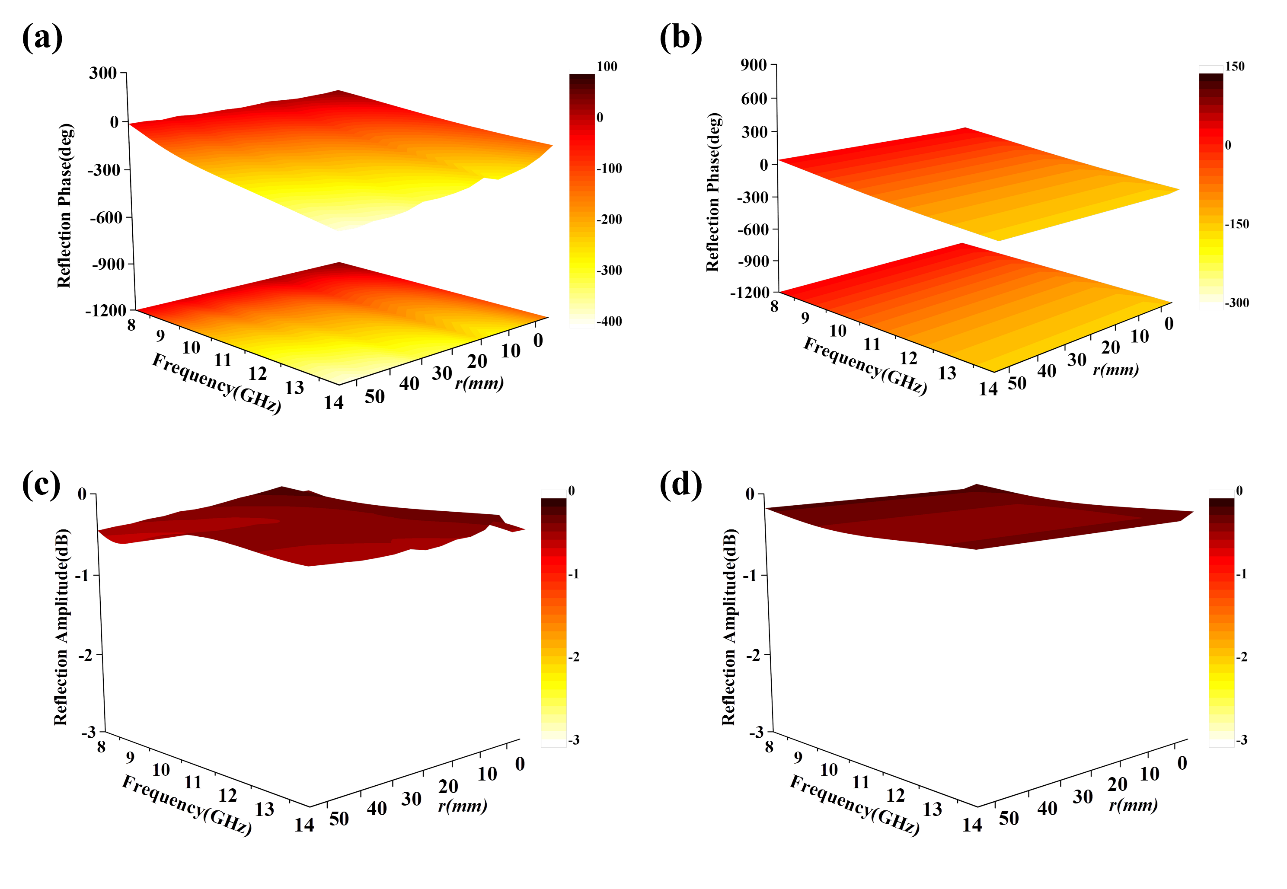
**

**Figure S4.** Simulation results of the distribution of reflection characteristics for the FAM, filled with different fluids. The reflection phase **(a)** and reflection amplitude **(c)** for the LM inside the FAM, and the reflection phase **(b)** and reflection amplitude **(d)** for the NaOH solution inside the FAM.

Supplementary Note 6: Optimization and Validation of Encoding Threshold Parameters

In this section, we provide a comprehensive comparative analysis to justify the selection of the encoding threshold parameter ($\eta=0.3$). It is essential to first clarify the operational relationship between the FAM platform and the encoding algorithm. The FAM platform, composed of 25 distributed sub-arrays, is engineered to faithfully execute any given binary encoding map. Therefore, the core objective is to determine which digital encoding strategy best represents the electromagnetic signature of the phantom target before it is physically mapped onto the metasurface.

To address this, we analyzed the camouflage effects for the aircraft illusion under three distinct threshold conditions: $\eta=0.2$, 0.3, and 0.4. We conducted a detailed morphological comparison of the synthesized electric field intensity distributions corresponding to these thresholds.


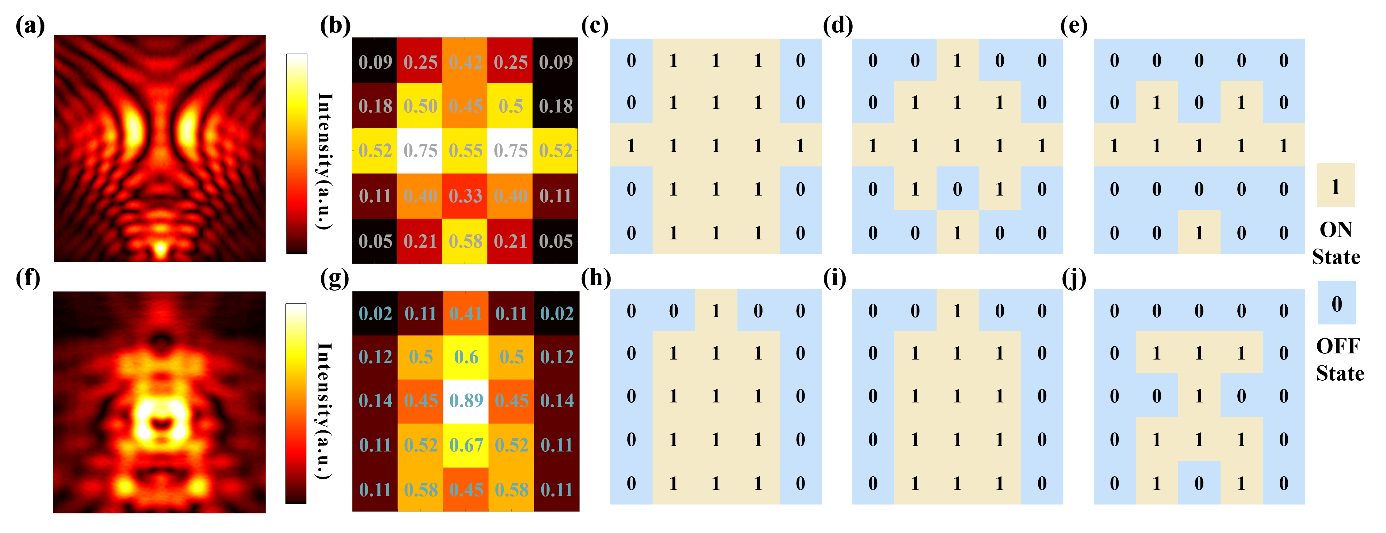


**Figure S5.** Comparative analysis of encoding generation strategies for aircraft and tank illusions. **a,f)** Simulated near-field electric field distributions of the ideal aircraft and tank models, serving as the ground truth targets. **b,g)** Normalized mean electric field intensity distributions mapped onto the 25 discrete sub-array regions of the FAM, representing the discretized analog signatures. **c,h)** Generated binary encoding patterns with a threshold of $\eta=0.2$. **d,i)** Generated binary encoding patterns with the optimal threshold of $\eta=0.3$. e,j) Generated binary encoding patterns with a threshold of $\eta=0.4$.

As illustrated in **Figure S5**, the electric field distribution reveals critical differences in fidelity and structural integrity across the different thresholds:

Low Threshold ($\eta=0.2$): This setting results in an over-saturated electric field distribution. Due to the low filtering criterion, an excessive number of supercells are activated. The resulting field pattern exhibits "bloating," where the sharp contours of the aircraft are expanded and blurred. This redundancy introduces unnecessary background scattering noise, which degrades the contrast and precision of the intended illusion.

High Threshold ($\eta=0.4$): This setting leads to feature erosion. The high filtering criterion is overly selective, causing the suppression of weaker but structurally important scattering centers. The resulting electric field distribution appears fragmented, with discontinuities in the wing and tail sections of the aircraft. This loss of secondary scattering features results in an incomplete electromagnetic signature that may fail to be recognized as the specific target.

Intermediate Threshold ($\eta=0.3$): This setting achieves the optimal reconstruction of the electromagnetic profile. It successfully balances the suppression of background noise with the preservation of critical geometric details. The electric field distribution under this threshold maintains the continuous contour of the target while accurately reproducing the intensity gradients of the primary scattering centers.

Consequently, based on the morphological fidelity of the reconstructed electric field, $\eta=0.3$ was selected as the optimal parameter. This ensures that the FAM generates a radar signature that is physically robust and visually consistent with the intended phantom target.

Supplementary Note 7: Characterization of Background Field Non-uniformity in the Large-Area FAM Prototype

As discussed in the main text, a key challenge in experimentally characterizing any electrically large metasurface is managing inherent system-level non-idealities. To visualize and quantify these effects, a baseline measurement of the FAM prototype in its uniform "all-OFF" state, where all microchannels were filled with NaOH solution, was performed.

The resulting near-field electric energy distributions across the 9-14 GHz bandwidth are presented in **Figure S6**. These maps clearly and consistently deviate from an ideal flat plane, exhibiting a complex spatial non-uniformity with a peak-to-trough variation of up to 7-8 dB. This non-uniformity is an expected consequence of the experimental realities associated with large-aperture measurements. It is primarily attributed to the non-uniform illumination profile of the feed horn over the large 555 mm * 500 mm metasurface, as well as the unavoidable assembly tolerances of the tiled array, such as minor planar deviations at the panel seams. We also note that the required vertical orientation of the FAM during testing likely exacerbates these geometric imperfections due to gravitational stress.

This characterization confirms that the background field, while non-uniform, represents a static and repeatable signature of our specific experimental system. Therefore, this non-ideal baseline precludes a direct comparison of absolute field values with idealized simulations but serves as the crucial reference against which the relative performance of the FAM's programmed states should be assessed.


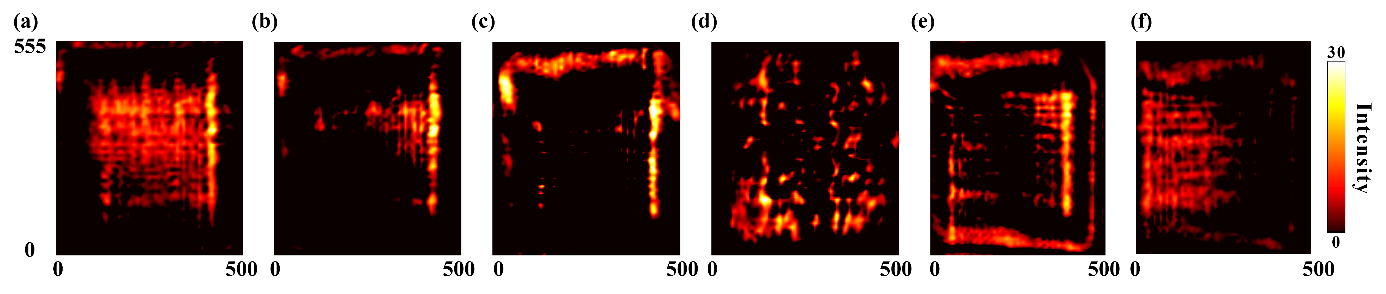


Figure S6. Near-field characterization of the FAM in a uniform 'all-OFF' state across the operational bandwidth. a-f) The simulated/measured near-field electric energy distributions are shown from 9 GHz to 14 GHz. A complex, non-flat background response is consistently observed across all frequencies, with significant spatial intensity variations.

Supplementary Note 8: Microfluidic Design, Infusion Protocol, and Stability Analysis

This detailed note provides a comprehensive description of the microfluidic engineering strategies employed to ensure the precise, stable, and repeatable reconfiguration of the Fluidic-Accessible Metasurface. The discussion encompasses the multiphysics-guided geometric optimization at the meta-atom level, the specific fluidic infusion protocol at the supercell level, the experimental validation of actuation reliability, and the resulting system-level programmability.

1. Multiphysics-Guided Geometric Design (Meta-atom Level)

The successful and repeatable operation of the FAM platform hinges on a robust and multi-scale microfluidic design. The foundation of this design lies at the meta-atom level where achieving stable and complete fluidic switching is critical. To overcome the common issues associated with traditional microfluidic designs such as unstable filling, capillary breakup, and liquid residue, we conducted a systematic optimization process using COMSOL Multiphysics. This rigorous analysis allowed us to co-optimize the two-phase flow characteristics and their direct impact on the electromagnetic properties.

We performed a comparative analysis of several distinct microchannel structures to evaluate their fluidic performance. Among the candidates, the design designated as Microchannel III was identified as the optimal geometry. The simulation results demonstrated that this specific structure ensures a stable flow interface with no residue formation thereby enabling the perfect realization of the targeted electromagnetic characteristics. In contrast, other geometries were prone to pinning effects or incomplete displacement which would degrade the RF performance.

Furthermore, we systematically studied the effects of key geometric and operational parameters to ensure reliability under varying conditions. Specifically, the influence of channel width and flow velocity on filling stability was analyzed to establish a robust operational window. By mapping the stability boundaries, we ensured that the surface tension and viscous forces remain balanced during actuation. The corresponding simulation results for different channel designs and the parametric studies regarding flow stability are presented in **Figure S8**. These results guide the fabrication process to guarantee that every meta-atom in the array operates within the stable flow regime.


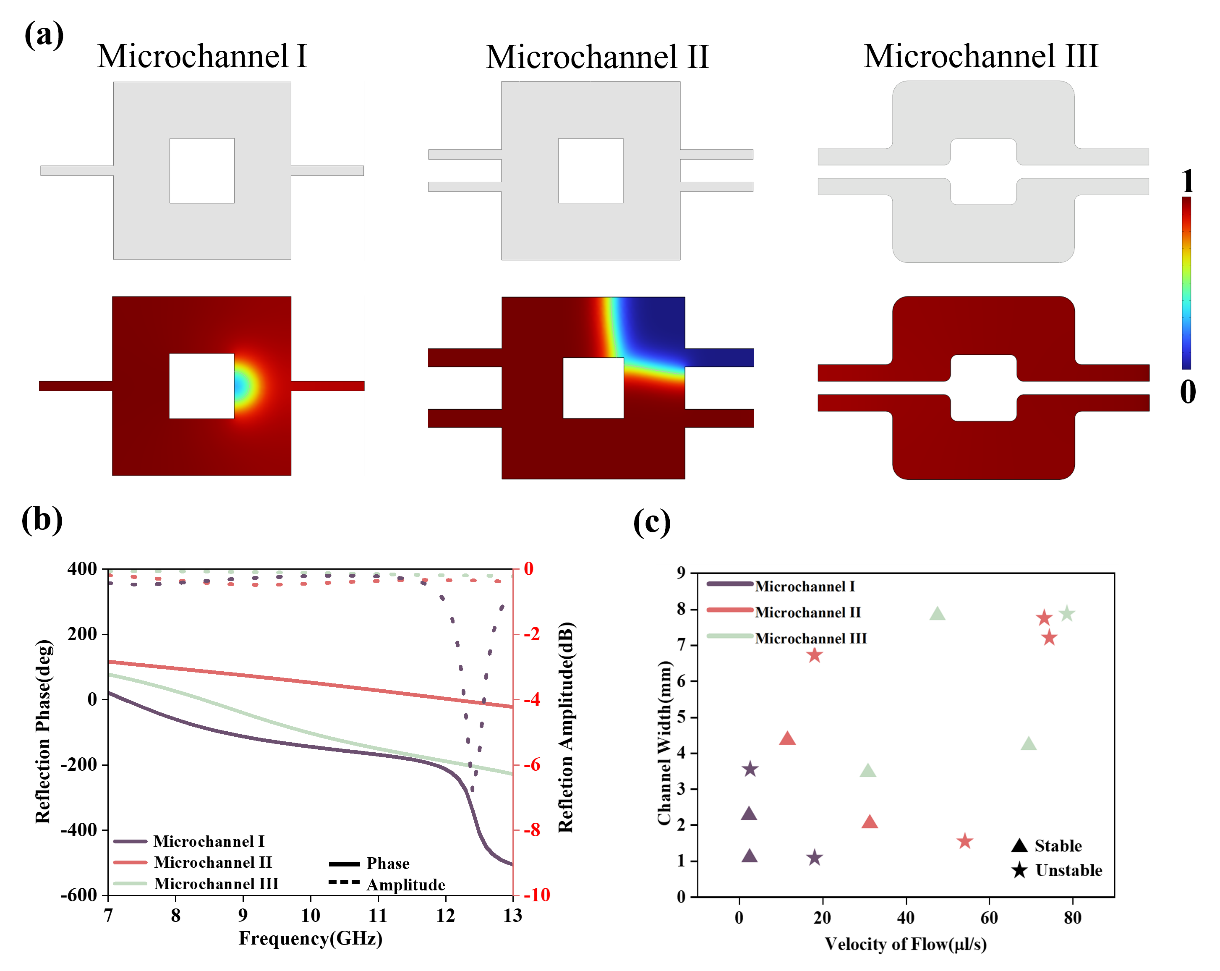


**Figure S7.** Fluid characteristics analysis of quasi-three-dimensional microfluidic meta-atom. **a)** The flow response of different microchannel structures and their internal LM and NaOH solution two-phase microfluidic flow. **b)** Comparison of LM residues inside unstable microfluidic structures and two-phase microfluidics inside stable microfluidics. **c)** The stability of two-phase microfluidic flow in different microfluidic structures under different flow channels and flow rates.

2. Fluidic Infusion and Encapsulation Protocol (Supercell Level)

Building upon this optimized meta-atom geometry, we established a comprehensive infusion protocol at the supercell level to ensure stable and long-term performance. A key aspect of this protocol is a fluidic encapsulation strategy utilizing a sandwich structure which is schematically detailed in **Figure S8.**

As illustrated in **Figure S8a**, the system utilizes a slug flow regime where a core segment of LM is strictly confined between a leading NaOH plug and a trailing NaOH plug. This configuration effectively isolates the LM from any residual air within the intricate channels of the supercell which prevents oxidation and the formation of a rigid oxide skin. A critical feature of this design is the formation of a continuous slip layer or lubrication film between the LM and the channel walls. This thin film of NaOH solution serves dual critical functions. First, it chemically dissolves the gallium oxide skin via a reduction reaction to maintain the LM in a highly mobile and spherical state. Second, it physically minimizes friction to prevent residue adhesion during movement.

**Figure S8b** further demonstrates the dynamic switching mechanism between the dielectric state filled with NaOH and the metallic state filled with LM. To ensure reliable external actuation, the fluidic ports of the supercell are interfaced with the external pumping system using detachable Luer connectors. These standardized connectors provide a secure and high-pressure seal preventing accidental disconnection and ensuring the safety and repeatability of the reconfiguration process for each supercell.


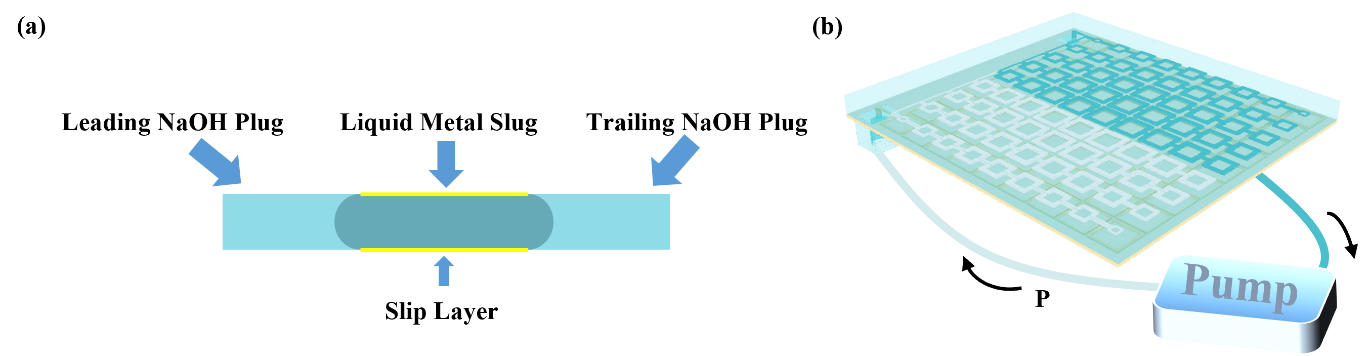


**Figure S8. Schematic of the fluidic actuation principle.** **a**) The sandwich encapsulation structure showing the LM slug bounded by leading and trailing NaOH plugs. A thin slip layer of NaOH solution forms between the metal and the channel wall to ensure residue-free motion. **b**) The dynamic displacement switching mechanism illustrating the reversible transition between State 0 where the supercell is filled with NaOH solution for the dielectric mode and State 1 where the LM displaces the solution to establish the metallic mode under pneumatic pressure.

3. Experimental Validation of Cycle Reliability

To rigorously validate the effectiveness of the encapsulation protocol and the geometric optimization, we performed continuous cyclic actuation tests on the fabricated supercell spanning 50 complete cycles. As presented in the newly added **Figure S9**, we captured optical images of the device's filled states at four representative milestones corresponding to the 5th, 10th, 25th, and 50th injection cycles. These visual records provide direct evidence of the structural consistency of the LM patterns throughout the test duration.

Specifically, the images confirm that the LM slug achieves robust filling of the resonant cavities. It is worth noting that as the number of cycles increases, minor incomplete filling was observed in the extreme corners of a limited number of supercells. However, electromagnetic analysis confirms that these corner regions are not the dominant distribution areas for the induced surface currents. Consequently, these slight geometric deviations have a negligible impact on the overall resonant response and electromagnetic performance of the FAM.

Overall, the structural integrity of the LM geometry remains highly stable throughout the testing period. This consistency in the conductive pattern validates that the proposed FAM maintains reliable electromagnetic functionality for practical long-term applications.


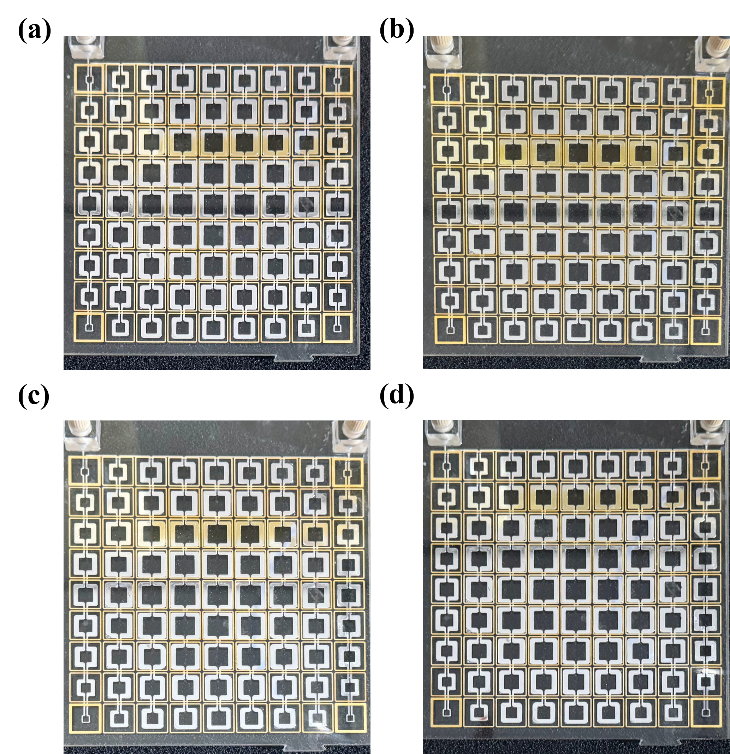


**Figure S9. Visual verification of fluidic stability and repeatability.** Optical images of the FAM supercell captured after the 5th, 10th, 25th, and 50th complete reconfiguration cycles. The images confirm that the LM slug achieves homogeneous filling without fragmentation during injection and leaves no visible residue upon withdrawal. These results demonstrate the robustness of the anti-oxidation and encapsulation strategies, ensuring reliable performance over long-term operation.

4. System-Level Programmability

Ultimately, this multi-scale design methodology grants us precise and repeatable control over the spatial distribution of LM and NaOH within each supercell which directly translates into electromagnetic programmability. By selectively programming the state of each supercell, we can create arbitrary and user-defined encoding patterns on the metasurface. This capability to program the scattering signature of the aperture supercell by supercell is what enables the FAM to achieve dynamic and convincing electromagnetic illusions for various complex targets. The fluidic logic and the consequent electromagnetic response are visualized in Supplementary Video S1 which demonstrates the real-time operation of the platform.

1. N. Engheta, Metamaterials: Physics and Engineering Explorations, Wiley-Interscience, Hoboken, N.J 2006.
2. S. Liu, J. Zhang, J. Wang, Y. Jia, Y. Liu, S. Gong, Q. Guo, P. Li, *Laser Photonics Rev*, 2025, 2500327.
